# *Sinorhizobium meliloti* succinylated high‐molecular‐weight succinoglycan and the *Medicago truncatula* LysM receptor‐like kinase MtLYK10 participate independently in symbiotic infection

**DOI:** 10.1111/tpj.14625

**Published:** 2020-01-11

**Authors:** Fabienne Maillet, Joëlle Fournier, Hajeewaka C. Mendis, Million Tadege, Jiangqi Wen, Pascal Ratet, Kirankumar S. Mysore, Clare Gough, Kathryn M. Jones

**Affiliations:** ^1^ LIPM Université de Toulouse, INRA, CNRS Castanet‐Tolosan CS 52627 France; ^2^ Department of Biological Science Florida State University Tallahassee FL 32306 USA; ^3^ Department of Plant and Soil Sciences Institute for Agricultural Biosciences Oklahoma State University Ardmore OK 73401 USA; ^4^ Noble Research Institute LLC. 2510 Sam Noble Parkway Ardmore OK 73401 USA; ^5^ IPS2 Institute of Plant Sciences Paris-Saclay IPS2, CNRS, INRA, Université Paris-Sud, Université Evry, Université Paris-Saclay Bâtiment 630 91405 Orsay France; ^6^ Institute of Plant Sciences Paris-Saclay IPS2, Paris Diderot, Sorbonne Paris-Cité Bâtiment 630 91405 Orsay France

**Keywords:** beneficial microbes, microbiome, exopolysaccharide, infection thread, LysM‐receptor‐like kinase, *Medicago truncatula*, root hair, *Sinorhizobium meliloti*, succinoglycan, symbiotic nitrogen fixation

## Abstract

The formation of nitrogen‐fixing nodules on legume hosts is a finely tuned process involving many components of both symbiotic partners. Production of the exopolysaccharide succinoglycan by the nitrogen‐fixing bacterium *Sinorhizobium meliloti* 1021 is needed for an effective symbiosis with *Medicago* spp., and the succinyl modification to this polysaccharide is critical. However, it is not known when succinoglycan intervenes in the symbiotic process, and it is not known whether the plant lysin‐motif receptor‐like kinase MtLYK10 intervenes in recognition of succinoglycan, as might be inferred from work on the *Lotus japonicus* MtLYK10 ortholog, LjEPR3. We studied the symbiotic infection phenotypes of *S. meliloti* mutants deficient in succinoglycan production or producing modified succinoglycan, in wild‐type *Medicago truncatula* plants and in *Mtlyk10* mutant plants. On wild‐type plants, *S. meliloti* strains producing no succinoglycan or only unsuccinylated succinoglycan still induced nodule primordia and epidermal infections, but further progression of the symbiotic process was blocked. These *S. meliloti* mutants induced a more severe infection phenotype on *Mtlyk10* mutant plants. Nodulation by succinoglycan‐defective strains was achieved by *in trans* rescue with a Nod factor‐deficient *S. meliloti* mutant. While the Nod factor‐deficient strain was always more abundant inside nodules, the succinoglycan‐deficient strain was more efficient than the strain producing only unsuccinylated succinoglycan. Together, these data show that succinylated succinoglycan is essential for infection thread formation in *M. truncatula*, and that MtLYK10 plays an important, but different role in this symbiotic process. These data also suggest that succinoglycan is more important than Nod factors for bacterial survival inside nodules.

## Significance Statement

This work should be of interest across the field of plant–microbe endosymbioses by providing insights into key determinants of both bacteria and plants for successful microbial host invasion. It is timely with much current interest in symbiotic roles of plant LysM‐RLK proteins and the evolutionary origins of nitrogen‐fixing endosymbiosis. MtLYK10 is a *Medicago truncatula* component that specifically intervenes in rhizobial infection, independently of succinoglycan recognition.

## Introduction

*Sinorhizobium meliloti* 1021 is a nitrogen‐fixing symbiont of the plants *Medicago truncatula* cv. Jemalong, *M. truncatula* ssp. *tricycla* R108, and *Medicago sativa* (alfalfa) (Hoffmann *et al.*, 1997; Oldroyd *et al.*, 2011). On roots of compatible hosts, *S. meliloti* induces nodule organogenesis, and invades and colonizes the developing nodule primordia, ultimately being endocytosed by cells of the nodule cortex (Oldroyd *et al.*, 2011). Once internalized within so‐called ‘symbiosomes’, the bacteria differentiate into the nitrogen‐fixing ‘bacteroid’ form (Vasse *et al.*, 1990), and fix dinitrogen gas, converting it to ammonia and providing it to the host (Hellriegel and Wilfarth, 1888; Burris, 1974). Invasion of host roots by rhizobia requires that compatible symbiont/host pairs exchange multiple signals that promote bacterial entry. *S. meliloti* produces a lipo‐chitooligosaccharide signal called Nod factor (NF) (Peters *et al.*, 1986; Lerouge *et al.*, 1990) responsible for inducing root hair curling around attached rhizobia. This traps *S. meliloti* microcolonies within an infection chamber (Fournier *et al.*, 2015). NFs also induce cell division in the root cortex leading to formation of nodule primordia (Timmers *et al.*, 1999; Xiao *et al.*, 2014). Rhizobia access root cortical cell layers through structures called infection threads that initiate from the colonized curled root hairs (CCRHs). An infection thread is a progressive ingrowth of root hair cell membrane that forms a tube populated with *S. meliloti* and filled with a matrix of bacterial exopolysaccharide (EPS), secreted bacterial proteins, and plant cell wall material (Brewin, 2004; Gage, 2004). *S. meliloti* propagate in the infection thread during its extension, a process that requires bacterial production of both NFs and the EPS succinoglycan (Jones *et al.*, 2007). Infection threads then extend through each successive cell layer until they reach the inner cortex, where bacteria are endocytosed by proliferating cells of the nodule primordium (Timmers *et al.*, 1999; Xiao *et al.*, 2014).

On the host *M. truncatula*, *S. meliloti* succinoglycan is required for symbiosis and cannot be substituted by other polysaccharides produced by this bacterium (Glazebrook and Walker, 1989). It is needed for infection thread progression on *M. truncatula* A17 (Jones *et al.*, 2008) and on alfalfa (Cheng and Walker, 1998). Both *M. truncatula* A17 (Jones *et al.*, 2008) and alfalfa (Niehaus *et al.*, 1993) show signs of plant defense when inoculated with the succinoglycan‐deficient *exoY* mutant. Succinoglycan is a polymer of an octasaccharide composed of seven glucose and one galactose sugar that *S. meliloti* produces in both high molecular weight (HMW) and low‐molecular‐weight (LMW) forms (Reinhold *et al.*, 1994; González *et al.*, 1998). Production of the LMW form is dependent on cleavage by the glycanases encoded by the *exoK* and *exsH* genes (York and Walker, 1997; Mendis *et al.*, 2016). Succinyl, pyruvyl, and acetyl groups are added to *S. meliloti* succinoglycan by, respectively, the products of the *exoH*, *exoV*, and *exoZ* genes (Reuber and Walker, 1993).

Not only is there a requirement for succinoglycan for host plant invasion, but the material that is produced must be properly modified by succinylation. If succinoglycan is not succinylated by ExoH, no functional nodules are formed by *S. meliloti* 1021 on *M. truncatula* A17 (Mendis *et al.*, 2016) or on alfalfa (Leigh *et al.*, 1987). Unsuccinylated succinoglycan produced by *exoH* mutants cannot be cleaved by *exoK‐* or *exsH‐*encoded glycanases, leaving all the unsuccinylated polymer produced by *exoH* mutants in the HMW form (York and Walker, 1998). We determined that the strict requirement for the succinylated form of succinoglycan for functional nodule formation on *M. truncatula* A17 is independent of the effect that succinylation has on production of the LMW form by the ExoK and ExsH glycanases (Mendis *et al.*, 2016). *S. meliloti* strains lacking both glycanases can form a successful symbiosis on *M. truncatula* A17 despite producing only HMW succinoglycan. However, these ‘double‐glycanase mutants’ have reduced symbiotic efficiency relative to control strains (*c.* 70% shoot fresh weight of wild‐type (WT)‐inoculated plants) (Mendis *et al.*, 2016). Strikingly, strains that lack the *exoH‐*encoded succinyltransferase but are otherwise isogenic to the *exoKdel/exsH* double‐glycanase strains, have the same plant growth phenotype as non‐inoculated plants or plants inoculated with the succinoglycan‐deficient *exoY* mutant (*c.* 18% of shoot fresh weight of *S. meliloti* WT‐inoculated plants (Mendis *et al.*, 2016)). Thus, loss of the LMW form of succinoglycan results in reduction of symbiotic efficiency, but the HMW form plays an even more critical role in the symbiosis as long as it is succinylated.

We set out to further characterize the symbiotic phenotypes of *exoY* succinoglycan‐deficient and *exoH* succinyltransferase‐deficient *S. meliloti* mutants. Specifically, to determine if these strains are equally deficient in invasion, and whether the defect occurs at the same stage in infection. We also aimed to determine how these rhizobial mutants and the LMW succinoglycan‐deficient strains interact with *M. truncatula* plants carrying a mutation in a LysM‐receptor‐like kinase (LysM‐RLK), MtLYK10 (Medtr5g033490). This plant protein is the ortholog of the exopolysaccharide receptor 3 (EPR3) protein from the determinate‐nodule‐forming legume *Lotus japonicus* (Buendia *et al.*, 2018). In the symbiotic interaction between the *L. japonicus* ecotype 'Gifu' and its symbiont *Mesorhizobium loti* R7A, *M. loti* EPS facilitates more efficient infection thread formation and nodulation, but EPS is not strictly required for symbiosis (Kelly *et al.*, 2013). *Ljepr3* null mutants permit invasion of *M. loti exoU* mutants that produce a truncated, pentasaccharide form of EPS that blocks *M. loti* invasion on WT plants (Kawaharada *et al.*, 2015). This suggests that LjEPR3 plays a role in EPS recognition and restricts infection of bacteria that produce inappropriate EPS (Kawaharada *et al.*, 2015).

We identified a *M. truncatula* mutant in *MtLYK10*, and determined how this line interacts with WT *S. meliloti* and *S. meliloti* mutants that are completely succinoglycan deficient, those that do not produce LMW succinoglycan and those that produce only unsuccinylated HMW succinoglycan. Our results suggest that *S. meliloti* succinoglycan has a function in infection of *M. truncatula* that is independent of *LYK10*, but is entirely dependent on succinylation of the polysaccharide.

## Results

### *Sinorhizobium meliloti* succinoglycan‐deficient and succinoglycan‐succinyltransferase‐deficient mutants form arrested infection threads in both *M. truncatula* cv. Jemalong and *M. truncatula* ssp. *tricycla* R108 ecotypes

Analyses of infection threads formed by *S. meliloti exo* mutants have previously been performed in alfalfa cv. Iroquois (Leigh *et al.*, 1987; Yang *et al.*, 1992; Cheng and Walker, 1998), but the symbiosis between *S. meliloti* 1021 and *M. truncatula* has important differences from the symbiosis with alfalfa. Increased production of succinoglycan by *S. meliloti* 1021 enhances infection on *M. truncatula* Jemalong, but not on alfalfa (Jones, 2012). A second EPS (called EPSII or galactoglucan) produced by some strains of *S. meliloti* (but not *S. meliloti* 1021) can function in place of succinoglycan on alfalfa cv. Iroquois (González *et al.*, 1996; Pellock *et al.*, 2000), but not on *M. truncatula* (Glazebrook and Walker, 1989). Indeed in a similar comparison performed in our growth conditions, we confirmed that EPSII cannot substitute for succinoglycan on *M. truncatula* cv. Jemalong A17 (Figure S1). Therefore, we first examined infection threads formed on *M. truncatula* by *S. meliloti exo* mutants to determine how the different types of *exo* mutants are impaired in invasion.

**Figure 1:**
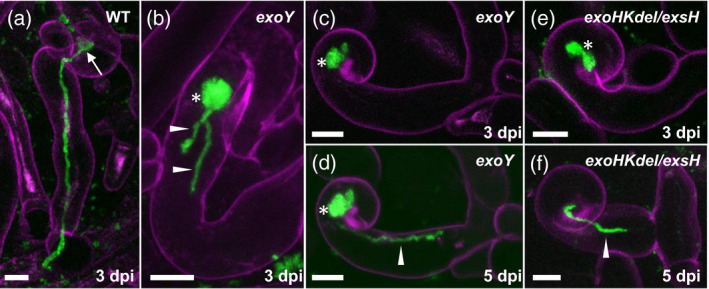
Infections formed by the succinoglycan‐deficient *exoY* mutant and the *exoHKdel/exsH‐1345* mutant on *Medicago truncatula* Jemalong *sunn‐2*. (a) WT *Sinorhizobium meliloti* 1021 expressing cCFP in a fully extended infection thread formed at 3 dpi on *M. truncatula* Jemalong *sunn‐2*. (b–d) CCRHs and short epidermal infection threads formed by the *S. meliloti exoY* mutant also expressing cCFP at 3 dpi (b, c) or 5 dpi (d). Note that in (c) and (d) the same site was imaged at two time‐points. (e, f) Infections formed by the *exoHKdel/exsH‐*1345 mutant at 3 dpi (e) or 5 dpi (f). Short, aborted infection threads (b, d, f) are labeled with arrowheads. The microcolony (infection chamber) is indicated in (a) (arrow). Excessively large root hair microcolonies (b–e) are labeled with asterisks. Images in (a–f) are z‐projections of confocal image stacks, combining the cCFP fluorescence of bacteria (green) and root hair cell wall autofluorescence (magenta). Results presented are representative of seven infection sites for *S. meliloti* WT 1021 (a), 24 sites for *S. meliloti exoY* (b–d) and 31 sites for *S. meliloti exoHKdel/exsH‐*1345 (e, f) recorded on three plants (six roots), five plants (11 roots) and seven plants (11 roots) respectively. Scale bars: 10 μm.

In *M. truncatula* A17, plants inoculated with an *exoY* mutant or with *S. meliloti* 1021 WT look similar at 3 days post‐inoculation (dpi), with *exoY*‐inoculated plants not yet having produced the large numbers of failed infection threads that will accumulate over time (Jones *et al.*, 2008). (See Table 1 and Figure S2 for strain descriptions.) However, using cerulean Cyan Fluorescent Protein (cCFP)‐expressing bacteria at the very low culture density of OD_600_ = 0.001, the near‐absence of background fluorescence makes it possible to see subtle differences in infection threads between *exoY*‐inoculated and WT‐inoculated roots. On *M. truncatula* Jemalong *super numeric nodules‐2* mutant *(sunn‐2*) inoculated with *S. meliloti* 1021 WT expressing cCFP, infection threads can be observed at 3–5 dpi extended all the way to the base of a root hair cell (Figure 1a). (The *M. truncatula sunn‐2* mutant is routinely used for infection thread analysis because it produces more infection events without any qualitative differences compared with *M. truncatula* WT plants (Schnabel *et al.*, 2005, Fournier *et al.*, 2015)). In contrast with WT *S. meliloti* 1021, the *S. meliloti exoY* mutant at the same time point has formed only short, probably aborted infection threads (Figure 1b–d single arrowheads). The microcolony‐containing infection chambers formed by *exoY* within the CCRHs are much larger than those usually formed by WT bacteria (Figure 1b–d, asterisks).

**Table 1:** *Sinorhizobium meliloti* strains and plasmids*

Strain name	Promoter controlling *exoLAMON* operon	Production of succinylated succinoglycan	Production of LMW succinoglycan	Successful symbiosis with *M. truncatula*	Reference
*S. meliloti* 1021	native	+	+	+	Meade *et al. *(1982)
*exoY*	native	N/A	no succinoglycan	−	Cheng and Walker (1998)
modified wild‐type 961	*trp*	+	+	+	Mendis *et al. *(2013)
*exsH* 1317 modified wild‐type with mutation in *exsH* glycanase	*trp*	+	+	+	Mendis *et al. *(2016)
*exoKdel/exsH* 1325	*trp*	+	−	+, *c.* 70% shoot fresh weight of wild‐type	Mendis *et al. *(2016)
*exoHKdel/exsH* 1345	*trp*	−	−	−	Mendis *et al. *(2016)
*nodC* (Rm5613)	native	+	+	−	Jacobs *et al. *(1985)

Plasmid	Description	Reference
pXLGD4	*hemA‐lacZ* reporter plasmid, carrying tetracycline resistance (TcR)	Leong *et al. *(1985)
pHC60	pHC41 constitutively expressing green fluorescent protein(GFP) with the S65T mutation, TcR	Cheng and Walker (1998)
pcCFP	pHC60 derivative constitutively expressing Cerulean cyan fluorescent protein (CFP), TcR	Fournier *et al. *(2015))

* Strains and plasmids presented in Figures 1, 2, 3, 4, 5, 6, 7 are shown in this table. A table of all strains and plasmids discussed in the manuscript, including those only in the Supporting Information, is shown in Figure S2.

We next investigated the infection thread phenotypes of an *S. meliloti* strain that forms only HMW unsuccinylated succinoglycan. To this aim, we used the non‐polar *exoHKdel/exsH‐*1345 triple mutant whose symbiotic phenotype mainly results from the lack of the ExoH succinyltransferase (see next paragraph). In *M. truncatula sunn‐2* at 3 dpi, the *exoHKdel/exsH‐*1345 mutant has formed only excessively large CCRHs (Figure 1e) or aborted infection threads (Figure 1f). Thus, the infection threads formed by *exoY* and *exoHKdel/exsH*‐1345 on the *M. truncatula sunn‐2* line are quite similar in that they initiate some tubular growth but then elongate slowly and struggle to progress. No differences were observed in the infection thread phenotypes of these *exo* mutants on *M. truncatula sunn‐2* versus *M. truncatula* Jemalong A17, and infections initiated by *exoY* or *exoHKdel/exsH‐*1345 are never observed penetrating a nodule primordium in *M. truncatula* Jemalong A17 (Figure S4). Note that the isogenic *exoHKdel/exsH* mutants 1345, 1343, and 1349 are lacking function of not only the *exoH* succinyltransferase, but also of the glycanases encoded by *exoK* and *exsH* (Figure S2). Complementation with a plasmid carrying *exoH* returns these strains to the level of symbiotic function of a non‐polar *exoK* mutant (70% shoot fresh weight of WT) (Figure S5).

**Figure 2:**
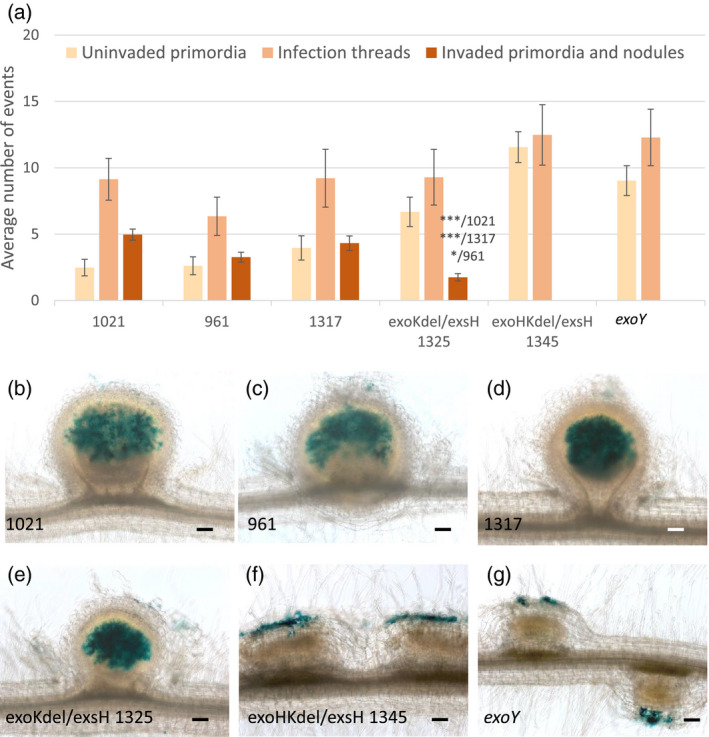
Infection and nodulation invasion phenotypes of the succinoglycan‐deficient *exoY* mutant and the *exoHKdel/exsH‐*1345 mutant on *Medicago truncatula* ssp. *tricycla* R108. Infection and nodule invasion phenotypes of *M. truncatula* WT R108 plants were scored 17 dpi following inoculation with six different *Sinorhizobium meliloti* strains; 1021, 961, *exsH‐*1317, *exoKdel/exsH‐*1325, *exoHKdel/exsH‐*1345 and *exoY*. The first three of these strains correspond to the unmodified WT strain (1021), a modified WT strain (961), which serves as a control for the expression of *exoLAMON* genes from a heterologous promoter, and *exsH*‐1317, which is identical to 961 except it lacks the *exsH*‐encoded glycanase. All three strains display an identical symbiotic phenotype. The double‐glycanase *exoKdel/exsH‐*1325 mutant (deficient for both the *exoK* and *exsH* glycanases) makes only HMW succinoglycan. The *exoHKdel/exsH‐*1345 mutant is isogenic to *exoKdel/exsH‐*1325, except that 1345 is missing the *exoH* succinyltransferase, and thus makes non‐succinylated HMW succinoglycan. *exoY* is completely deficient for production of succinoglycan. (a) Quantification of uninvaded nodule primordia, infection threads, and invaded nodule primordia and nodules. Data are the averages of two independent experiments, *n* = 28 for each strain. Error bars = SEM. Statistically significant differences in invaded primordia and nodules between *exoKdel/exsH‐*1325 and other strains are indicated. *P < 0.05; ***P < 0.001. (b–g) Photographs illustrating these infection phenotypes, all inoculated strains carrying a constitutive *hemA‐lacZ* reporter gene fusion (pXLGD4) for visualization of bacteria in blue. Fully invaded nodules were seen with 1021, 961, *exsH*‐1317 and *exoKdelexsH*‐1325 (b–e, respectively), while only uninvaded nodule primordia were observed with *exoHKdel/exsH‐*1345 and *exoY* (f and g, respectively). In these latter two cases, rhizobia appear restricted to the epidermis, but apparently continue to multiply in these unsuccessful infection compartments, resulting in excessively large root hair microcolonies. Scale bars: 100 µm.

*exo* mutant infection thread development and nodule formation were also analyzed on the host *M. truncatula* ssp. *tricycla* R108. This is the genotype for most *Tnt1* retrotransposon‐induced plant mutants available for *M. truncatula* (d'Erfurth *et al.*, 2003; Tadege *et al.*, 2008). Two *S. meliloti* strains, 961 and *exsH*‐1317, which have the *exoLAMON* operon transcribed from a heterologous promoter and retain the WT copy of the *exoH* and *exoK* genes (Figures S2 and S3) were first tested. The *exoLAMON* operon has been placed under heterologous regulation in these strains to isolate it from polar effects from mutations in the upstream *exoH* and *exoK* (Mendis *et al.*, 2013; Mendis *et al.*, 2016). The control strains have the same altered genetic regulation as the *exoK* and *exoHK* deletion strains (Figures S2 and S3). Both strains form fully invaded nodules by 17 dpi on *M. truncatula* R108, similarly to WT *S. meliloti* 1021 (Figure 2). In addition, the double‐glycanase *exoKdel/exsH*‐1325 mutant, which only makes HMW succinoglycan (Mendis *et al.*, 2016) also forms invaded nodules on *M. truncatula* R108, although significantly fewer nodules form compared with inoculation with the two WT 1021, 961 or single mutant *exsH‐*1317 strains (Figure 2). This is consistent with the fact that inoculation with the double‐glycanase *exoKdel/exsH*‐1325 mutant ultimately produces plants with *c.* 70% the shoot fresh weight of those inoculated with control strains (Mendis *et al.*, 2016). In contrast with these strains, neither the *S. meliloti exoHKdel/exsH‐*1345 mutant nor the *S. meliloti exoY* mutant forms any invaded primordia or nodules on *M. truncatula* R108 (Figure 2). Instead, both strains have an arrested infection thread phenotype on *M. truncatula* R108 that closely resembles the phenotype on *M. truncatula* Jemalong. Thus, at 17 dpi, both *exoHKdel/exsH‐*1345 and *exoY* form excessively large microcolonies in CCRHs, and only very short infection threads that are blocked within root hairs and/or in the outer cortical cell layer above uninvaded nodule primordia (Figure 2). These infection blocks presumably result in the arrest of nodule development, such that only nodule primordia are formed. The complete lack of successful infection of nodule primordia explains the absence of shoot fresh weight gain by either strain (Mendis *et al.*, 2016).

### *M. truncatula lyk10* mutant plants show an early infection phenotype with WT *S. meliloti*

Having established these phenotypes on *M. truncatula* R108 plants, we studied an R108 line with a *Tnt1* insertion in the *LjEPR3* ortholog *MtLYK10* (Medtr5g033490) (Cheng *et al.*, 2014). Phylogenetic and synteny analysis confirmed the orthology between *MtLYK10* and *LjEPR3*, and revealed that the genomes of some plants, including some Fabaceae species, encode two *MtLYK10* homologs, but not *M. truncatula* (Figure S6). qRT‐PCR analysis confirmed that the *Tnt1* insertion results in a null mutation in *MtLYK10* (Figure S7).

**Figure 3:**
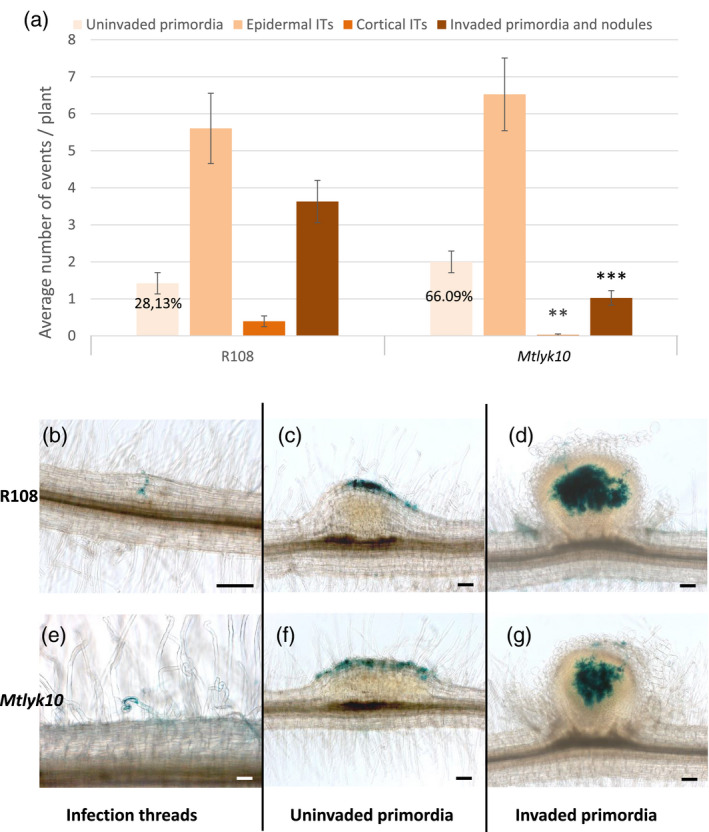
Early rhizobial infection and nodulation invasion phenotypes of *Medicago truncatula lyk10* mutant plants with *Sinorhizobium meliloti* WT. Infection and nodulation invasion phenotypes of *M. truncatula Mtlyk10* mutant and R108 WT plants were scored 11 dpi following inoculation with the *S. meliloti* WT strain 1021. (a) Quantification of uninvaded nodule primordia, epidermal (restricted to the epidermis) and cortical (which have reached the cortex) infection threads (ITs) and invaded nodule primordia and nodules. Data are the averages of two independent experiments, *n* = 38 for each genotype. Error bars = SEM. On *Mtlyk10* plants compared with R108, significantly fewer cortical infection threads, and significantly fewer invaded primordia and nodules formed. **P < 0.01; ***P < 0.001. The number of uninvaded primordia formed was not significantly different (*P* = 0.0962) between the two genotypes, but 28.13% (for R108) and 66.09% (for *Mtlyk10*) of all nodules and primordia were uninfected, as indicated. (b–g) Photographs illustrating these infection phenotypes, the inoculated strain carrying a constitutive *hemA‐lacZ* reporter gene fusion (pXLGD4) for visualization of bacteria in blue. (b) a cortical infection thread in R108; (c) an uninvaded nodule primordium in R108; (d) an infected nodule in R108; (e) an epidermal infection thread in *Mtlyk10*; (f) an uninvaded nodule primordium in *Mtlyk10*; (g) an infected nodule in *Mtlyk10*. Scale bars: 100 µm.

**Figure 4:**
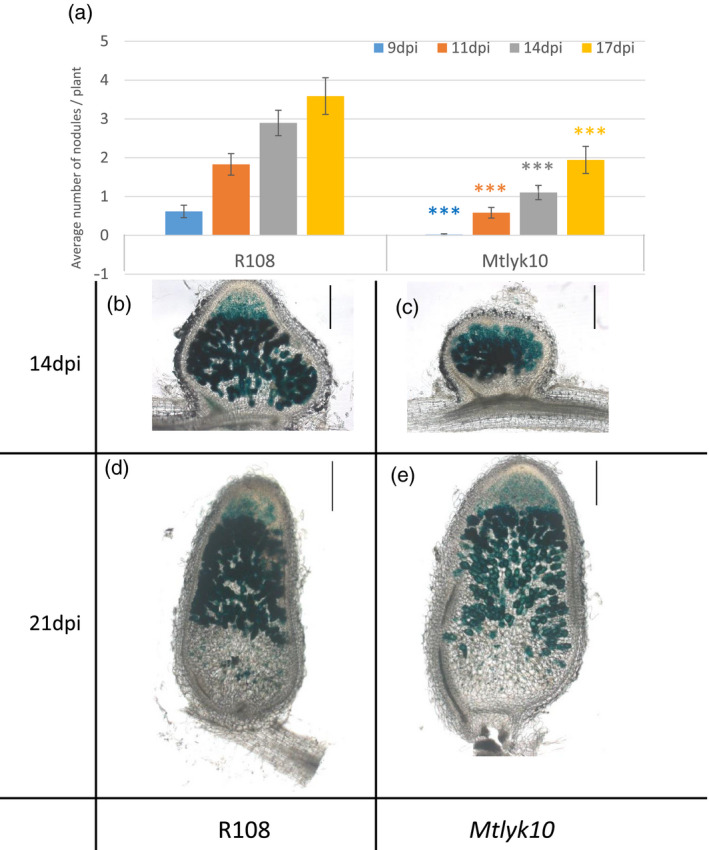
Kinetics of nodule appearance and invasion of well developed nodules on *Medicago truncatula lyk10* mutant plants following inoculation with *Sinorhizobium meliloti* WT. (a) The appearance of nodules was scored at 9, 11, 14 and 17 dpi following inoculation of *M. truncatula Mtlyk10* mutant and R108 WT plants with the *S. meliloti* WT strain 1021. *n* = 35 for each genotype. ***P < 0.001. Error bars = SEM. (b–e) Photographs of 70 μm nodule sections, colored to visualize bacteria in blue (the inoculated strain carries a constitutive *hemA‐lacZ* reporter gene fusion (pXLGD4)) after sectioning. (b) R108 at 14 dpi; (c) *Mtlyk10* at 14 dpi; (d) R108 at 21 dpi; (e) *Mtlyk10* at 21 dpi. Scale bars: 100 μm.

WT *S. meliloti* 1021 was able to form infection threads with a normal appearance on *Mtlyk10* mutant plants. However, compared with WT *M. truncatula* R108 plants, most infection threads remained in epidermal cells on *Mtlyk10* mutant roots and significantly fewer infection threads progressed into the cortex at 11 dpi with WT *S. meliloti* 1021. Consequently, significantly fewer successfully invaded nodule primordia formed on mutant roots (Figure 3a–g). Numbers of uninvaded nodule primordia (Figure 3a) were not significantly different between genotypes (P = 0.0962), and more were seen on R108 compared with previous work on A17. However, a higher proportion of all nodules/primordia were uninvaded for *Mtlyk10* (66.09%) compared with R108 (28.13%) (Figure 3a). Young nodules of *Mtlyk10* plants appeared normal and well‐invaded (Figure 3d,g). We also found that nodulation was delayed on *Mtlyk10*, and that more developed nodules are also equivalently well‐invaded in mutant and WT plants (Figure 4).

We also compared the nodulation ability of two independent lines carrying the *Mtlyk10* mutation, selected from the backcross to WT R108 plants. Both *Mtlyk10* lines similarly formed fewer invaded primordia and nodules, and fewer cortical infection threads than WT plants (Figure S8). We also showed that the infection and nodulation phenotype of a homozygous WT line selected from the F2 population of the backcross between the *Mtlyk10* mutant and R108 was comparable to the R108 WT line (Figure S8). Taken together, these data show that MtLYK10 plays a role in the symbiotic process of rhizobial infection thread growth, and by consequence the rate of successful nodulation in *M. truncatula*.

### *M. truncatula lyk10* mutant plants show more severe infection phenotypes with *S. meliloti* succinoglycan mutants

**Figure 5:**
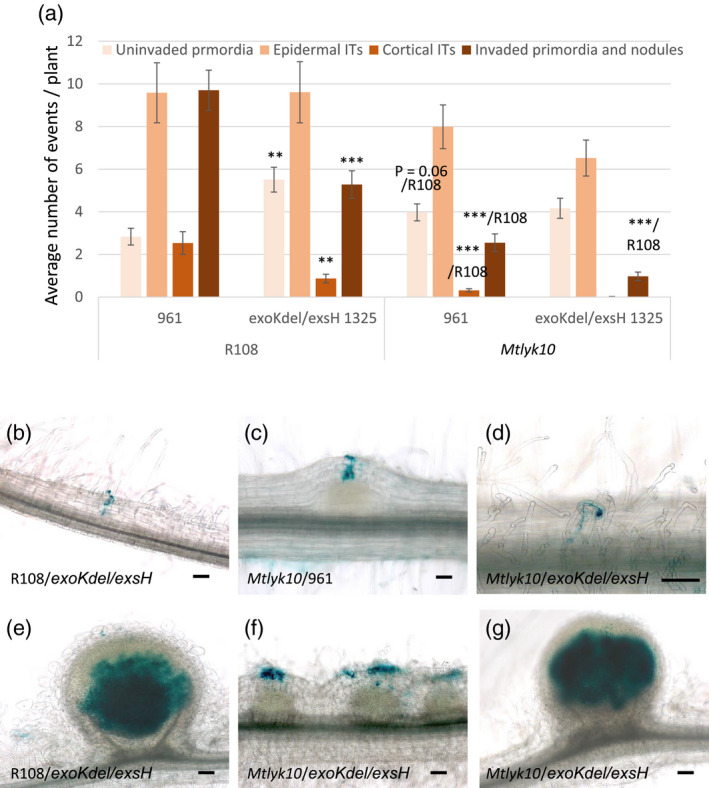
Infection and nodulation invasion phenotypes of the *exoKexsH* 1325 double‐glycanase *Sinorhizobium meliloti* mutant on *M. truncatula lyk10* mutant plants. Infection and nodulation invasion phenotypes of *M. truncatula Mtlyk10* mutant and R108 WT plants were scored 14 dpi following inoculation with the *exoKdel/exsH*‐1325 mutant that makes only HMW succinoglycan, or the *S. meliloti* WT strain 961. (a) Quantification of uninvaded nodule primordia, epidermal (restricted to the epidermis) and cortical (which have reached the cortex) infection threads (ITs), and invaded nodule primordia and nodules. Data are the averages of four independent experiments, *n* = 60 for each genotype/strain combination. Error bars = SEM. Statistically significant differences are indicated as ***/R108 for comparisons for the same rhizobial strain (either 961 or *exoKdelexsH*‐1325) between plant genotypes (R108 versus *Mtlyk10*); and as ** or *** for comparisons between the two rhizobial strains (961 versus *exoKdelexsH*‐1325) for the same plant genotype (R108). **P < 0.01; ***P < 0.001. No long infection threads were observed with *exoKdel/exsH*‐1325 on *Mtlyk10* plants. For the comparison of R108 and *Mtlyk10* plants inoculated by *exoKdel/exsH*‐1325, no significant difference was observed (P = 0.06) for numbers of uninvaded nodule primordia (as indicated). (b–g) Photographs illustrating these infection phenotypes, both inoculated strains carrying a constitutive *hemA‐lacZ* reporter gene fusion (pXLGD4) for visualization of bacteria in blue. (b) A cortical infection thread on R108 inoculated by *exoKdel/exsH*‐1325. (c) A cortical infection thread on *Mtlyk10* inoculated by 961. (d) An epidermal (restricted to the root hair) infection thread on *Mtlyk10* inoculated by *exoKdel/exsH*‐1325. (e) A nodule on R108 inoculated by *exoKdel/exsH*‐1325. (f) Uninvaded primordia on *Mtlyk10* inoculated by *exoKdel/exsH*‐1325. (g) A nodule on *Mtlyk10* inoculated by *exoKdel/exsH*‐1325. Scale bars = 100 µm except for (d) = 20 µm.

To address the possible implication of MtLYK10 in EPS recognition, we next characterized the symbiotic phenotypes of *Mtlyk10* mutant plants with *S. meliloti exo* mutants. First, we compared the double‐glycanase *exoKdel/exsH*‐1325 mutant (only makes HMW succinoglycan) with the appropriate modified WT *S. meliloti* 961 strain (makes both HMW and LMW succinoglycan), on *Mtlyk10* and R108 plants at 14 dpi. As we had observed with the unmodified 1021 WT *S. meliloti* (Figure 3), the 961 WT *S. meliloti* strain induced fewer cortical infection threads and fewer successful infections on *Mtlyk10* mutant plants compared with R108 plants (Figure 5). Also, no significant quantitative differences were noted in uninvaded nodule primordia (*P* = 0.06) induced by the 961 strain on *Mtlyk10* mutant and WT plants (Figure 5), as previously observed with *S. meliloti* 1021.

Comparing the infection phenotype of the *exoKdel/exsH*‐1325 mutant on *Mtlyk10* and WT roots, we observed no cortical infection threads on *Mtlyk10* roots and significantly fewer invaded nodule primordia and nodules on *Mtlyk10* roots compared with R108 (Figure 5). The different infection phenotypes are apparent in Figure 5(b) that shows an infection thread in the cortex with *exoKdel/exsH‐*1325/R108, Figure 5(c) that shows cortical infection with 961/*Mtlyk10*, and Figure 5(d) that shows an infection thread restricted to a root hair with *exoKdel/exsH‐*1325/*Mtlyk10*. As *S. meliloti exoKdel/exsH‐*1325 only makes HMW succinoglycan, this suggests that while LMW succinoglycan is not essential for infection, it enhances infection success in both R108 WT and *Mtlyk10* mutant plants. Furthermore, the absence of LMW succinoglycan together with an *MtLYK10* mutation creates a stronger infection defect than the absence of LMW succinoglycan alone.

**Figure 6:**
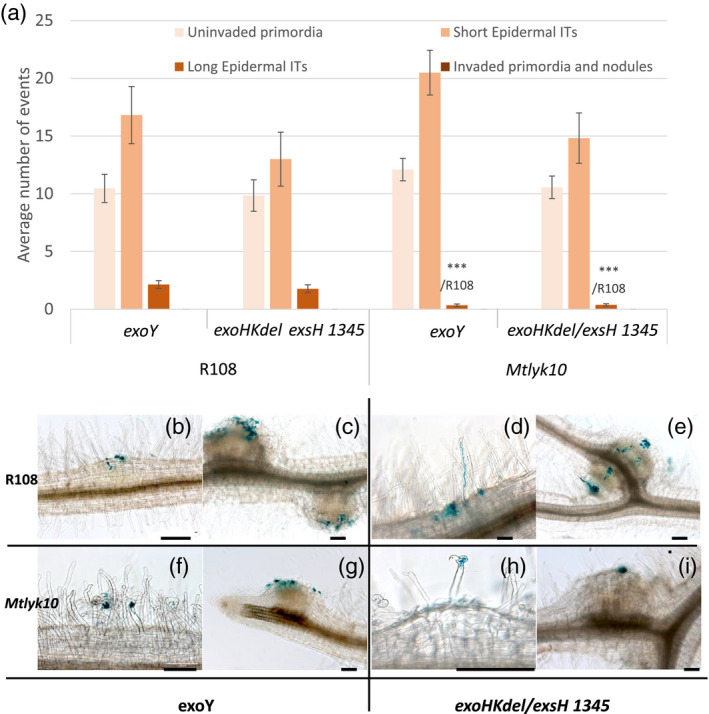
Infection and nodulation invasion phenotypes of the *Sinorhizobium meliloti* succinoglycan‐deficient *exoY* mutant and the *exoHKdel/exsH*‐1345 succinyltransferase mutant on *M. truncatula lyk10* mutant plants. Infection and nodulation invasion phenotypes of *M. truncatula Mtlyk10* mutant and R108 WT plants were scored 21 dpi following inoculation with the *S. meliloti* succinoglycan‐deficient *exoY* mutant or the *S. meliloti exoHKdel/exsH‐*1345 mutant, which makes non‐succinylated HMW succinoglycan. (a) Quantification of uninvaded nodule primordia, and short and long epidermal infection threads. No invaded nodule primordia or invaded nodules were seen. Data are the averages of two independent experiments, *n* = 40 for each genotype/strain combination. Error bars = SEM. Statistically significant differences are indicated as ***/R108 to indicate that the comparison is to the R108 genotype inoculated with the same rhizobial strain. ***P < 0.001. (b–g) Photographs illustrating these infection phenotypes, both inoculated strains carrying a constitutive *hemA‐lacZ* reporter gene fusion (pXLGD4) for visualization of bacteria in blue. (b, c) R108/*exoY*; (d, e) R108/*exoHKdel/exsH‐*1345; (f, g) *Mtlyk10*/*exoY*; (h, i) *Mtlyk10*/*exoHKdel/exsH‐*1345. Scale bars = 100 µm.

WT R108 and *Mtlyk10* mutant plants were next inoculated with succinoglycan‐deficient *S. meliloti exoY*, and the *S. meliloti exoHKdel/exsH‐*1345 mutant that only produces non‐succinylated HMW succinoglycan. In order to assess both infection and nodulation, we observed plants at 21 dpi. As previously shown (Figure 2) neither strain successfully infects R108, and in addition, we observed no successful infections with either strain on *Mtlyk10* plants (Figure 6a–i). Indeed, on *Mtlyk10* mutant plants both strains induced mainly uninfected bumps corresponding to uninvaded nodule primordia with large, abnormal colonies of rhizobia that remain superficial to these structures (Figure 6g,i). These infection events were scored as short epidermal infection threads (Figure 6a), illustrated in Figure 6(f) (*exoY/Mtlyk10*) and Figure 6(h) (*exoHKdel/exsH‐*1345*/Mtlyk10)*. Some more developed, but nonetheless also aborted, infection threads (‘long epidermal infection threads’) were observed with both strains on WT R108 plants (for example, Figure 6d shows a long epidermal infection thread for *exoHKdel/exsH‐*1345/R108), but significantly fewer of such long, aborted infection threads were observed on *Mtlyk10* mutant roots compared with WT R108, with both *exo* mutants (Figure 6a). Thus, the severe infection phenotype of *exoY* and *exoHKdel/exsH‐*1345 on WT plants was even stronger on *Mtlyk10* mutant plants. Taken together, none of the rhizobial mutant phenotypes were suppressed by the mutation in *MtLYK10*, and in fact, the phenotypes were worse with each of the three *S. meliloti exo* mutants (*exoKdel/exsH* 1325, *exoY* and *exoHKdel/exsH‐*1345).

### Rescue *in trans* by a strain producing normal succinoglycan indicates that succinoglycan‐defective mutants are at a disadvantage in mixed inoculation nodules, with non‐succinylated succinoglycan being even more of a disadvantage than the absence of succinoglycan

Rescue of failed infection by succinoglycan‐deficient *exo* mutants has previously been accomplished by providing WT succinoglycan by co‐inoculation with NF‐deficient mutants (Klein *et al.*, 1988). Each strain individually is impaired in invasion, but when NF and succinoglycan are provided *in trans* within an infection thread, both strains succeed in invading. We used this *in trans* rescue method to investigate whether the non‐succinylated succinoglycan produced by *exoHKdel/exsH‐*1345 has a restrictive effect on the invasion of a strain that produces normal succinoglycan. To determine if a NF‐deficient *S. meliloti* mutant rescues the *exoHKdel/exsH*‐1345 mutant with the same efficiency with which it rescues the succinoglycan‐deficient *exoY* mutant, we performed co‐inoculations with a mutant in the NF N‐acetylglucosaminyltransferase gene, *nodC* (Jacobs *et al.*, 1985; Atkinson and Long, 1992). If the *nodC* mutant rescues *exoHKdel/exsH‐*1345 less efficiently than it rescues *exoY*, it suggests that any restrictive interaction between non‐succinylated succinoglycan and the plant is dominant over the reaction with WT succinoglycan. If *nodC* rescues *exoHKdel/exsH‐*1345 equal to or more efficiently than it rescues *exoY*, it suggests that any restrictive interaction is not dominant.

**Figure 7:**
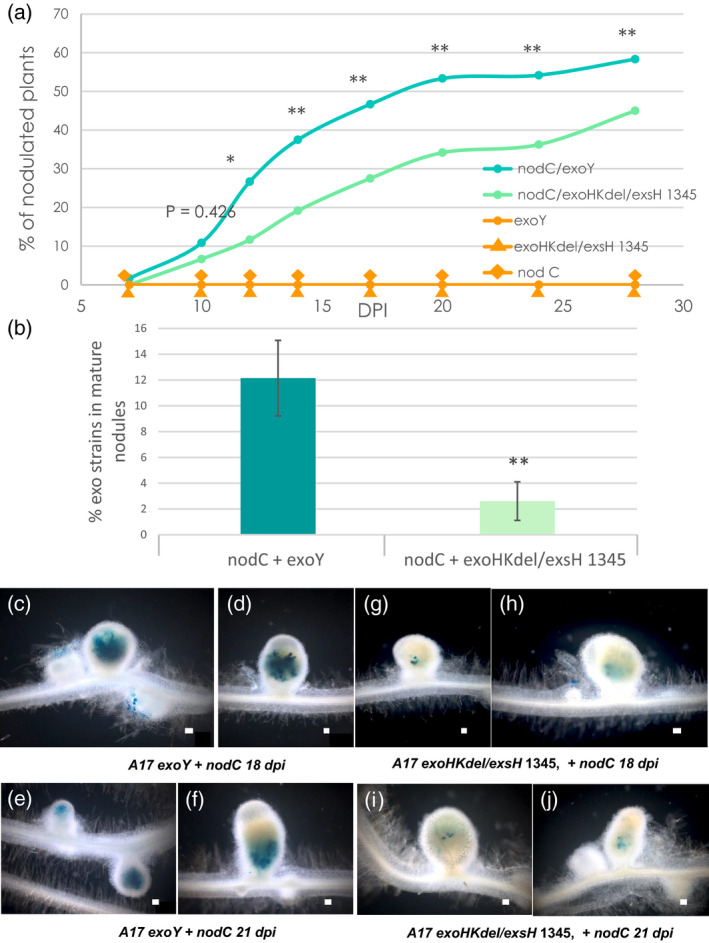
Nodulation defects of *exo* mutants can be rescued *in trans* by co‐inoculation with a *nodC* mutant. *M. truncatula* WT A17 plants were inoculated with 1:1 mixes of *Sinorhizobium meliloti nodC* + *exoY* or *S. meliloti nodC* + *exoHKdel/exsH‐*1345, or with individual control strains; *exoY*, *exoHKdel/exsH‐*1345 or *S. meliloti nodC*. (a) Kinetics of nodule formation at 7, 10, 12, 14, 17, 20, 24, and 28 dpi expressed as percentages of nodulated plants at each time point. Data are the averages of two independent experiments *n* = 120 for each mixed inoculum. Statistical comparisons were performed between the two types of mixed inoculation on the average number of nodules per plant at each time point. *P < 0.05; **P < 0.01. (b) Percentages of the *exoY* or *exoHKdel/exsH‐*1345 mutants recovered from mature nodules compared with total rhizobial counts recovered. Error bars = SEM. **P < 0.01. (c–j) Photographs of mature nodules formed in the same experimental set‐up using the *exoY* and *exoHKdel/exsH‐*1345 mutants carrying a constitutive *hemA‐lacZ* reporter gene fusion (pXLGD4) for visualization of bacteria in blue. (c–f) nodules formed following co‐inoculation with *S. meliloti* 1021 *nodC* + *exoY* at 18 dpi (c, d) and 21 dpi (e, f). (g–j) nodules formed following co‐inoculation with *S. meliloti* 1021 *nodC* + *exoHKdel/exsH‐*1345 at 18 dpi (g, h) and 21 dpi (i, j). Scale bars = 100 µm.

*M. truncatula* A17 WT plants were inoculated with 1:1 mixtures of either *nodC* + *exoY* or *nodC* + *exoHKdel/exsH‐*1345, and nodule formation tracked over 28 days. As shown in Figure 7(a) both combinations induce nodule formation, in contrast with the negative controls of individual strain inoculations. Compared with *nodC* + *exoY*, the *nodC* + *exoHKdel/exsH‐*1345 mix was less efficient, with fewer plants forming nodules. In both cases relatively few nodules were observed (<1 nodule/plant) (Figures 7 and S9). To determine strain occupancy, mature nodules were surface sterilized, then crushed and bacterial growth was quantified on antibiotic selection plates (for *nodC*) or on Calcofluor (for *exo* strains). This showed that both types of nodules contained a clear majority of *nodC* bacteria (90–95%), and that *exoY* was significantly more present in mixed inoculation nodules compared with *exoHKdel/exsH‐*1345 (Figure 7b).

To visualize nodule occupancy by the *exo* mutant strains, plants were co‐inoculated with the same mixes of *S. meliloti* strains, but using *exo* strain versions expressing beta‐galactosidase. Unlike for recoverable rhizobia, the *in situ* staining via *lacZ* of rhizobia inside nodules also enables visualization of terminally differentiated bacteria that are internalized within host cells. Coloration of well developed nodules of both types confirmed the presence of the *exo* strains in nodules, and again indicated that *exoHKdel/exsH‐*1345 is less present compared with *exoY* (Figure 7c–j). Thus, *nodC* + *exoY* nodules clearly showed the presence of *exoY*, usually throughout inner nodule tissue (Figure 7c–f). The dense staining of the *exoY* rhizobia within nodules suggests that they are internalized into cortical cells. In contrast, *nodC* + *exoHKdel/exsH‐*1345 nodules showed only quite limited, patchy areas of the *exoHKdel/exsH‐*1345 mutant within inner nodule tissue (Figure 7g–j). This suggests that the *exoHKdel/exsH‐*1345 mutant has a very limited, if any, ability to become terminally differentiated into bacteroids. In both cases, smaller nodule‐like structures that were visualized along with the mature nodules at 18–21 dpi, were not invaded by the *exo* strains. Co‐inoculation studies using R108 were only feasible in the presence of the ethylene biosynthesis inhibitor aminoethoxyvinylglycine (AVG) (extremely few nodules formed without AVG). Ethylene is a well known negative regulator of rhizobial infection, and symbiotic responses and nodulation are often better when AVG is present (Suzaki *et al.*, 2015). In these conditions, co‐inoculated R108 plants did not show clear differences in nodulation ability by the two mixes of rhizobial strains. Nonetheless, coloration of nodules clearly showed *exoHKdel/exsH‐*1345 was less persistent in mixed inoculation nodules compared with *exoY* (Figure S10), as observed on *M. truncatula* A17. Taken together, these data indicate that early events of infection by *trans‐*complementation of NF‐deficient and succinoglycan‐deficient/aberrant rhizobial mutants lead to fully formed nodules in which the main occupant is the strain with normal succinoglycan (*nodC*). Furthermore, the *exoHKdel/exsH‐*1345 mutant producing non‐succinylated succinoglycan appears at even more of a disadvantage than the succinoglycan‐deficient *exoY* strain in mixed inoculation nodules.

## Discussion

Our results show that the succinoglycan‐deficient *exoY* mutant of *S. meliloti* is completely blocked in invasion of *M. truncatula*, and only forms very short infection threads that do not elongate and do not reach beyond the epidermis. The *exoHKdel/exsH*‐1345 mutant that produces succinoglycan missing the succinyl group is as impaired in invasion as the succinoglycan‐deficient *exoY* mutant, with infections being similarly restricted to the epidermis. These infection phenotypes explain the previous finding that inoculation of *M. truncatula* A17 with either mutant results in the same plant growth phenotype as non‐inoculated plants (Jones *et al.*, 2008; Mendis *et al.*, 2016). Thus, no successful infections are possible without succinylated succinoglycan. Unlike these two non‐invading mutant strains, we had previously observed that the *S. meliloti exoKdel/exsH‐*1325 mutant, which only produces succinylated HMW succinoglycan, invades, but has a reduced symbiotic efficiency (Mendis *et al.*, 2016). In this study, we now show that the *S. meliloti exoKdel/exsH‐*1325 mutant forms fewer invaded nodules than WT strains, so the enhancement that production of the LMW form of succinoglycan provides to the symbiotic productivity of *S. meliloti* on the host *M. truncatula* is due to increased infection thread progression, which results in more nitrogen‐fixing nodules. Thus, succinylated succinoglycan plays a critical and early role in infection thread progression in *M. truncatula* root hairs.

Rhizobial NFs are also essential for the infection process in *M. truncatula*, as in the majority of rhizobial host plants (Dénarié *et al.*, 1996). Mutant *M. truncatula* plants expressing reduced levels of the *M. truncatula* NF receptor proteins MtNFP or MtLYK3 exhibit arrested infections with unusually large microcolonies of rhizobia and sac‐like infection events (Arrighi *et al.*, 2006; Smit *et al.*, 2007), which resemble to some extent, those we observed with *S. meliloti exoY* or *exoHKdel/exsH‐*1345. Thus, NF perception and the presence of succinylated succinoglycan are both needed for the correct initiation, maintenance, and direction of polar growth of infection threads, essential processes for successful infection of *M. truncatula*. In contrast with NFs, succinoglycan is not essential for root hair curling and rhizobial entrapment in root hair curls, neither for infection thread initiation nor the induction of nodule primordia formation, such that *S. meliloti exoY* or *exoHKdel/exsH‐*1345 mutants induce uninvaded nodule‐like structures, unlike NF‐deficient *S. meliloti* mutants (Dénarié *et al.*, 1996). This suggests that an early symbiotic role of succinoglycan is probably restricted to a local function for infection thread elongation, unlike the pleiotropic roles of NFs.

The rescue of both *S. meliloti exoY* and *exoHKdel/exsH‐*1345 by the NF‐deficient *nodC* mutant shows that the prevention of epidermal penetration by both the succinoglycan‐deficient *exoY* mutant and the *exoHKdel/exsH‐*1345 mutant is relieved when normal succinoglycan is supplied. However, complementation efficiency was relatively low, and low percentages of *exo* rhizobia were recovered from within mature nodules. This suggests that these mutants were both at a disadvantage compared with the *nodC* mutant in nodules, and that once nodules are formed, the rhizobial production of NFs is less important than the extracellular rhizobial succinoglycan. Alternatively, differences in the quantities of NFs and succinoglycan could be required during nodulation. Laser‐capture microdissection RNA‐Seq experiments show that *exo* and *nod* genes are expressed in apices and nitrogen‐fixing zones of *M. truncatula* nodules (Roux *et al.*, 2014). A disadvantage for internalization into and/or survival within symbiosomes relative to the *nodC* mutant might be due to greater sensitivity on the part of *exo* mutants to conditions within the symbiosome. As the dominance of *nodC* in *exoHKdel/exsH‐*1345 + *nodC* nodules (approximately 98%) was even more marked compared with *exoY* + *nodC* nodules (approximately 88%), we can hypothesize that the production of only non‐succinylated succinoglycan renders rhizobia even more sensitive than the complete absence of succinoglycan production. However, if the plant does actively resist invasion by strains producing structurally aberrant succinoglycan, this is only partially effective and the complete absence of succinoglycan is also resisted.

We have also shown that *M. truncatula* plants carrying a *Tnt1* insertion in *MtLYK10* have clear, early infection phenotypes, the severity of which depends on the *S. meliloti* strain tested. In response to WT rhizobia, *Mtlyk10* plants form fewer cortical, successful infection threads and consequently fewer invaded nodules, compared with WT plants. Although *Mtlyk10* mutant plants were clearly defective in infection, nodule primordia and some invaded nodules can be formed, indicating that the NF‐activated process of nodule organogenesis is independent of MtLYK10. *Mtlyk10* mutants are thus symbiotic *M. truncatula* mutants that can uncouple infection and nodule organogenesis (Murray, 2011), revealing another plant component that is specifically involved in the infection process in *M. truncatula*. The expression profile of *MtLYK10* is compatible with its role in infection, with strong induction in root hairs and nodules, and in response to either NFs or rhizobial inoculation, like many infection‐related genes (Breakspear *et al.*, 2014; Roux *et al.*, 2014; Larrainzar *et al.*, 2015; van Zeijl *et al.*, 2015; Damiani *et al.*, 2016; Jardinaud *et al.*, 2016; Liu *et al.*, 2019). Within *M. truncatula* nodules, *MtLYK10* is one of the most highly expressed LysM receptor genes, with strong expression in the distal infection zones of nodules, similarly to *MtNFP* and *MtLYK3* (Roux *et al.*, 2014; Bono *et al.*, 2018).

In response to the *S. meliloti exo* mutants studied here, the infection phenotype of *Mtlyk10* mutant plants was consistently more severe compared with *S. meliloti* WT strains on these plants. The *S. meliloti exoKdel/exsH‐*1325 mutant forms very few invaded nodules on *Mtlyk10* mutant plants, while no invaded nodules and very few extended infection threads were observed with either the *exoY* or the *exoHKdel/exsH‐*1345 strains. Thus, the infection deficiencies conferred by defects in succinoglycan or mutation in *MtLYK10* were apparently additive, indicating different mechanisms by which succinoglycan and MtLYK10 play a role in the infection process. Furthermore, if the infection deficiency of the *S. meliloti exoY* mutant is due to perception of bacterial molecular patterns exposed by the absence of succinoglycan, the restriction is not accomplished through MtLYK10.

In *L. japonicus,* the octasaccharide EPS produced by *M. loti* is also implicated in symbiosis. However, there are important differences between the two model nodulation systems in the role of EPS in infection thread formation. Notably, a *M. loti* R7A *exoB* mutant that does not make EPS can invade *L. japonicus*, albeit with reduced efficiency (Kelly *et al.*, 2013; Kawaharada *et al.*, 2015). Remarkably, in *M. loti*, structurally aberrant polysaccharide produced by the *exoU* mutant creates a much greater *L. japonicus* invasion defect than the complete absence of the EPS in an *exoB* mutant (Kelly *et al.*, 2013; Kawaharada *et al.*, 2015). It is not possible to perform a completely identical comparison between the two symbiont/host systems. There is no known mutant of *S. meliloti* that is comparable with the *exoU* mutant of *M. loti* in forming a truncated pentasaccharide EPS monomer. A glycosyltransferase that has been designated *exoU* in *S. meliloti* is known to function in the succinoglycan biosynthesis pathway, but this mutant does not secrete any succinoglycan (Reuber and Walker, 1993; Becker *et al.*, 1993c). We have confirmed that this mutant has a succinoglycan‐deficient phenotype similar to an *exoY* mutant, in terms of succinoglycan production (Figure S11). Nonetheless, we can conclude that the interaction between the *exoHKdel/exsH*‐1345 mutant and *M. truncatula* is different from the interaction between the *exoU* mutant of *M. loti* that produces truncated pentasaccharide EPS and *L. japonicus*.

Another major difference between the two systems is that *L. japonicus* mutants in the *MtLYK10* ortholog, *LjEPR3*, partially suppress the phenotype of a *M. loti exoU* mutant (Kawaharada *et al.*, 2015), while none of the *S. meliloti exo* phenotypes we have characterized here are suppressed by the *Mtlyk10* mutation. In fact, infection deficiencies are accumulated by the combination of plant (*Mtlyk10*) and rhizobial (*exoKdel/exsH‐*1325, *exoHKdel/exsH‐*1345 and *exoY*) mutations, indicating that each succinoglycan‐related infection phenotype is independent of MtLYK10. In other words, unlike the proposed role for *LjEPR3*, our data suggest that *S. meliloti* succinoglycan and MtLYK10 perform important, but different, roles in infection thread formation in *M. truncatula*. Thus, although we cannot exclude a role for MtLYK10 in recognizing a form of EPS not tested here, we favor the hypothesis that MtLYK10 does not intervene in EPS recognition. Despite the apparent difference between plant species for EPS recognition, *Ljepr3* and *Mtlyk10* mutants have similar phenotypes with WT rhizobia, both forming fewer invaded nodules than their corresponding WT plants, within 14 dpi. Also, these *Ljepr3* and *Mtlyk10* phenotypes are both weaker than those of the appropriate EPS‐minus rhizobial strains, *M. loti exoB* and *S. meliloti exoY*, respectively, on their WT host plants. This leaves open the possibility that an unknown EPS perception component exists in *L. japonicus*. So far, the few LysM‐RLKs that have been characterized in different plant species have mostly been linked to roles in recognizing chitin oligomers or lipo‐chitooligosaccharides, but as pointed out by Kawaharada *et al. *(2015), such proteins have LysM domains that are quite divergent from those of MtLYK10/LjEPR3 homologs. With the notable exception of *Arabidopsis thaliana* (Buendia *et al.*, 2018), these MtLYK10/LjEPR3 homologs are widespread in Angiosperms. Interestingly, some dicot species have two copies of this gene as the result of ancient duplications (Figure S6), followed potentially by neofunctionalization or subfunctionalization. This could point toward different functions of *MtLYK10*‐type genes in different plant species and this gene is not always involved in nodulation, as shown recently in *Parasponia andersonii* (Gough *et al.*, 2018; van Velzen *et al.*, 2018). This may be linked to differences in rhizobial infection mechanisms among plant species.

It is not yet clear what role(s) succinylated succinoglycan plays in infection thread development. *exoY*, *exoH*, and *exoK* transposon‐insertion mutants are sensitive to acidic pH (Hawkins *et al.*, 2017). However, acid sensitivity could not be the only cause of the symbiotic defect in these strains. *exoK* mutants are just as sensitive as *exoH* mutants to acid, yet the symbiotic defect of *exoK* mutants is much less severe than that of *exoH* mutants. There is some evidence for succinoglycan interaction with reactive oxygen species (ROS) produced in the infection thread. One possible role is protection of the bacteria from ROS. When tested in the free‐living state, succinoglycan‐overproducing strains are *c.* 1.5–2‐fold more resistant than WT *S. meliloti* to H_2_O_2_, whereas the *exoY* mutant has *c.* 0.4–0.5 the resistance to H_2_O_2_ of the WT (Lehman and Long, 2013). But the symbiotic phenotypes of *S. meliloti* mutants in genes involved in coping with reactive oxygen species do not closely resemble those of *exo* mutants. The extremely H_2_O_2_‐sensitive *S. meliloti oxyR* mutant has no symbiotic defect (Jamet *et al.*, 2005). Also, an *S. meliloti* double‐mutant in two catalase‐encoding genes *katB* and *katC* has a symbiotic defect, but in contrast with *exo* mutants, it forms numerous infection threads that do not appear to fail until the time of *S. meliloti* release into symbiosomes (Jamet *et al.*, 2003). Thus, it seems unlikely that sensitivity to ROS could be the only cause of the symbiotic defect of *exo* mutants.

Another recent article has shown that strains that produce an excess of succinoglycan are less sensitive to the NCR247 cationic peptide than is *S. meliloti* WT (Arnold *et al.*, 2018). However, the *exoY* mutant was found to not be more sensitive than the WT to NCR247, at least in the free‐living state, and addition of non‐succinylated succinoglycan was as successful as wild‐type succinoglycan in enhancing survival of free‐living *S. meliloti* treated with NCR247 (Arnold *et al.*, 2018). NCR peptides are secreted into the symbiosome by most legumes that form indeterminate nodules, including *M. truncatula* and alfalfa (Alunni and Gourion, 2016). These peptides play multiple roles in the process of bacteroid differentiation and in cell fate (Alunni and Gourion, 2016). A role has not yet been demonstrated for NCR peptides in root hairs and infection threads, and bacteria within *M. truncatula* infection threads do not show signs of bacteroid differentiation, but expression of NCR247 is strongly increased in root hairs by 3 dpi with *S. meliloti* (Breakspear *et al.*, 2014). A difference in sensitivity to NCR peptides might explain the disadvantage in nodule occupancy that the *exoY* and *exoHKdel/exsH‐*1345 mutants appear to have relative to the *nodC* mutant in our co‐inoculation experiments. More than 600 NCR genes are induced in *M. truncatula* by *S. meliloti*, and *in vitro* tests with NCR peptides show increased sensitivity and different responses of a *S. meliloti* succinoglycan‐deficient mutant compared with WT bacteria (Montiel *et al.*, 2017). Also, EPS‐minus mutants of *Sinorhizobium fredii* HH103 can form functional nodules on soybean and pigeonpea (Parada *et al.*, 2006), species which lack NCR genes (Montiel *et al.*, 2016), and also on *Glycyrrhiza uralensis* (Margaret‐Oliver *et al.*, 2012)*,* in which the expression of only a few NCR genes is detected (Montiel *et al.*, 2017). Taken together, these data suggest a link between rhizobial EPS and the mediation of NCR peptide effects on differentiation and survival of endophytic rhizobia at different stages of nodulation.

Thus, the essential function of succinylated succinoglycan in *Medicago* infection might involve interaction with an as‐yet‐unidentified plant receptor, or with another component of the infection thread matrix. Alternatively, succinylated succinoglycan could have a role in protecting the bacteria from NCR peptides or an as‐yet‐unidentified stress within the infection thread.

## Experimental Procedures

### Bacterial strains, growth conditions, and plasmids

*S. meliloti* 1021 strains (Meade *et al.*, 1982), were grown at 28–30°C with shaking in either Luria–Bertani Miller medium with 2.5 mm MgSO_4_ and 2.5 mm CaCl_2_ with streptomycin 200 or 500 µg/ml or glutamate–mannitol–salts (GMS) medium (York and Walker, 1997). *S. meliloti* mutants were grown in appropriate antibiotics at concentrations: neomycin 100 or 200 µg/ml; spectinomycin 50 µg/ml; gentamicin 25 µg/ml. For detail on strains, see Figure S2. Calcofluor polysaccharide indicator plates contained 0.02% Calcofluor white M2R (fluorescent brightener 28, Sigma, St. Louis, MO, USA) (Leigh *et al.*, 1985). Strains carrying plasmids: pRF771 (Wells and Long, 2002); pExoH (this study, see Figure S2); pXLGD4 (*hemA::lacZ* reporter (Leong *et al.*, 1985)); pHC60 (Cheng and Walker, 1998); or pcCFP (Fournier *et al.*, 2015) were selected with 10 µg/ml tetracycline.

### Plant genotypes

Two WT and two mutant genotypes of *Medicago truncatula* were used. *Medicago truncatula* cv. Jemalong A17 and *M. truncatula* ssp. *tricycla* R108 are WT lines. The *M. truncatula* Jemalong *super numeric nodules‐2* mutant *(sunn‐2*) carries a mutation in the *MtSUNN* gene, leading to loss of the autoregulation of nodulation and consequently to increased infection events (Schnabel *et al.*, 2005; Fournier *et al.*, 2008). The *M. truncatula* ssp. *tricycla Mtlyk10* (NF4929) mutant carries a *Tnt1* retrotransposon in LysM1 of the *MtLYK10* gene (Medtr5g033490). Two independent backcrossed lines of this mutant were used, most of the results presented were obtained using the *Mtlyk10#46* line (see Figure S8). A line that is homozygous for the WT *MtLYK10* gene was also selected after backcross and checked for its symbiotic phenotype (see Figure S8).

### Real time quantitative RT PCR analysis on *M. truncatula* plants

qRT‐PCR expression analysis of *MtLYK10* was performed on *M. truncatula* WT (R108) and the *Mtlyk10* mutant. Expression was analyzed in the non‐inoculated and nodulated root segments for each genotype, using material pooled from 20 plants for each genotype inoculated or not with *S. meliloti* 1021. Nodulated material was collected from 14 to 25 dpi. Relative expression was calculated using two housekeeping genes, Medtr3g062450 and Medtr3g065110, and then fold changes were calculated for inoculated compared with non‐inoculated as the 2^−ΔCT^ values. The primers for *MtLYK10* were: F: GACCCAGTAGCTGCTTTGGAA; and R: TGACACTGCCACAACGATCTC. The RNA extraction protocol and qRT‐PCR conditions and primers for the reference genes have been previously described (Gibelin‐Viala *et al.*, 2019).

### Whole‐plant assay of nodulation and symbiotic efficiency

*Medicago truncatula* cv. Jemalong A17 was prepared for inoculation with *S. meliloti* as previously described (Jones *et al.*, 2013). Seedlings were moved to individual Jensen’s medium microcosms and inoculated with *S. meliloti* of the appropriate strain. Plants were grown in a Percival AR‐36L incubator (Perry, IA) at 21°C, with 60–70% relative humidity and 100–175 µmol m^−2^ s^−1^ light. Shoot fresh weight was measured after 7 weeks.

### Infection thread assays of cCFP‐expressing *S. meliloti* strains on *M. truncatula* Jemalong

Rhizobial infection sites were analyzed in *M. truncatula* Jemalong *sunn‐2* or A17 root hairs by *in vivo* microscopy as previously described (Fournier *et al.*, 2008; Fournier *et al.*, 2015). In brief, plants were placed in 12 cm x 12 cm plates containing modified Fåhraeus medium (0.5% (w/v) Phytagel; Sigma‐Aldrich) supplemented with 50 nm aminoethoxyvinylglycine (AVG). Roots were covered with gas‐permeable plastic film (Lumox Film, Sarstedt, France), and plants were grown with the dishes slightly tilted to encourage the growth of the roots along the film. An aqueous suspension (OD_600_ = 0.001) of exponentially growing bacteria constitutively expressing cCFP was used for inoculation of nitrogen‐starved plants. Selected infection sites were imaged with a Leica TCS SP2 AOBS confocal laser scanning microscope (Figure 1) or a Zeiss epifluorescence microscope (Figure S4) equipped with long‐distance 40x HCX Apo L (numerical aperture, 0.80) water immersion objectives. For confocal images, the argon laser band of 458 nm was used to excite cCFP and a 561‐nm diode to observe cell wall autofluorescence, respectively. Specific emission windows used for cCFP and autofluorescence signals were 465–485 nm and 620–720 nm, respectively, and emitted fluorescence was false colored in green (cCFP) and magenta (wall autofluorescence). The composite images shown are maximal projections of selected planes of a *z*‐stack. For epifluorescence images, cCFP and cell wall autofluorescence were recorded using combinations of BP436/10 and BP450‐480 (for cCFP) or BP573/20 and BP625/30 (for autofluorescence) excitation and emission filters. Images were acquired and projected using Leica confocal software or MetaVue (Molecular Devices) and processed using Leica confocal software, metavue and fiji (http://imagej.nih.gov/ij/; Schindelin *et al.*, 2012; Schindelin *et al.*, 2015). Data are from two experiments in *sunn‐2* and 2 experiments in A17.

### Infection thread assays of β‐galactosidase‐expressing *S. meliloti* strains on *M. truncatula tricycla* R108

Seeds were surface sterilized, germinated, and grown in 12 cm x 12 cm plates containing slanting 0.2 mm NH_4_NO_3_‐Fåhraeus agar medium covered by growth paper for 6 days at 22°C with day/night cycles of 16/8 h. Each plant was inoculated with 2 × 10^3^ bacteria.

Entire roots were collected 11, 14, 17 or 21 dpi, fixed with 2% (v/v) glutaraldehyde for 2 h under vacuum, rinsed 3× in Z′ buffer [0.1 m potassium phosphate buffer (pH 7.4), 1 mm MgSO_4_, and 10 mm KCl], and stained 1 h under vacuum then overnight at 28°C in Z′ buffer containing 0.08% 5‐bromo‐4‐chloro‐3‐indolyl‐β‐d‐galactoside (X‐gal), 5 mm K_3_Fe(CN)_6_, and 5 mm K_4_Fe(CN)_6_. Entire roots were observed under a light microscope after 3 min bleach clearing (3.2% active chloride).

### Co‐inoculation assays

Plants grown as described above were inoculated with 100 µl per plant of a 1:1 mix of *nodC* + *exo* mutant at an OD_600_ = 0.5. Mature nodules were surface sterilized 5 min in bleach 2.4% active chloride, rinsed 5× in sterile water, crushed with a mortar in a 1.5 ml microtube, and spread on plates containing streptomycin and neomycin. Ten to 12 nodules of each combination were crushed and 52 randomly picked colonies/ nodule were characterized. *M. truncatula* A17 plants were grown without AVG, R108 plants were grown in the presence of 0.5 µm AVG.

### Statistical analysis

Data are presented as means ± SEM. Data analyses were performed with r software (v.3.4.1). Statistical comparisons of phenotypic data were performed using a Wilcoxon test or a Student’s *t*‐test (when the normality assumption was followed).

### Phylogeny analysis

MtLYK10 homologs were found from Buendia *et al. *(2018) and from NCBI blasts. Synteny was verified using phytozome (https://phytozome.jgi.doe.gov), soybase (https://www.soybase.org) and the Legume Information System, lis (http://legumeinfo.org). Protein or gene names are shown in the tree, together with the plant species name. The tree was generated using http://www.phylogeny.fr using the gblocks program to eliminate poorly aligned positions and divergent regions.

## Author Contributions

FM, JF, CG, KMJ designed research and wrote the manuscript. FM, JF, HCM, KMJ performed experiments. FM, JF, HCM, CG, KMJ analyzed data. MT, JW, PR, KM provided resources.

## Conflicts of interest

The authors have no conflicts of interest to declare.

## Supporting information


**Figure S1.** Plant performance and nodulation phenotypes on alfalfa cv. Iroquois and *M. truncatula* A17 of *S. meliloti* strains that make EPSII (galactoglucan).

**Figure S2.** Information for all *S. meliloti* strains and plasmids, both those are discussed in the main text and in Supporting Information.

**Figure S3.** Information on the genetic modifications in strains 961, 1317, *exoKdel/exsH*‐1325 and *exoHKdel/exsH‐*1345 with the *exoLAMON* operon under the control of a heterologous promoter.

**Figure S4.** Infections formed by the *exoY* and *exoHKdel/exsH‐*1345 mutants on *Medicago truncatula* Jemalong A17.

**Figure S5.** Complementation of strains with an *exoHK* deletion by a plasmid carrying *exoH*.

**Figure S6.** Phylogenetic tree of MtLYK10 homologs from a variety of plant species, mostly Rosid dicots, but also two monocots, *Oryza sativa* and *Brachypodium distachyon*, and with MtLYK11 as an outgroup.

**Figure S7.** qRT‐PCR expression analysis of *MtLYK10* in *Medicago truncatula* WT (R108) and the *Mtlyk10* mutant.

**Figure S8.** Comparison of the infection and nodulation phenotype of two independent *Mtlyk10* mutant lines (Mtlyk10#2 and Mtlyk10#46) and a homozygous *MtLYK10‐WT* line (MtLYK10‐WT), all selected from the backcross between the *Mtlyk10* mutant and R108.

**Figure S9.** Kinetics of nodule formation when the nodulation defects of *exo* mutants are rescued *in trans* by co‐inoculation with a *nodC* mutant.

**Figure S10.** Nodules formed after mixed inoculation on *Medicago truncatula* WT R108 plants.

**Figure S11.** The *exoU* mutant of *S. meliloti* does not produce detectable succinoglycan.

## Data Availability

Supporting Information is listed below and these figures are provided for this article. Image acquisition and modification information has been described in Experimental Procedures or in Figure legends.
