# Supplementary figures and images for: Sinorhizobium meliloti succinylated high‐molecular‐weight succinoglycan and the Medicago truncatula LysM receptor‐like kinase MtLYK10 participate independently in symbiotic infection
Source: Plant J. 2020 Jan 11;102(2):311–26. doi: 10.1111/tpj.14625 (PMC9327734; doi:10.1111/tpj.14625)

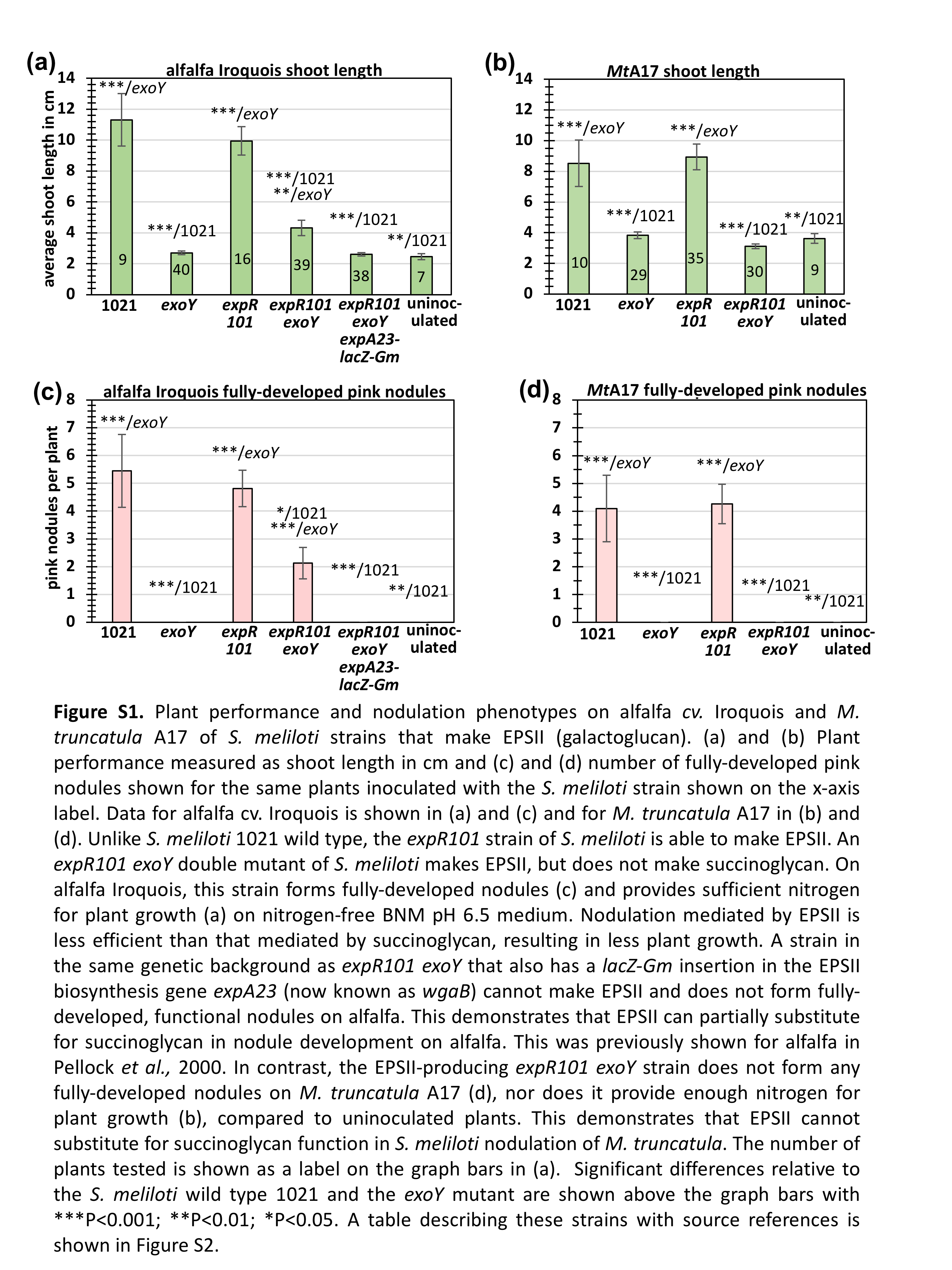

Supplement: Supplementary file 1 — Figure S1. Plant performance and nodulation phenotypes on alfalfa cv. Iroquois and M. truncatula A17 of S. meliloti strains that make EPSII (galactoglucan). [file TPJ-102-311-s006.tiff]

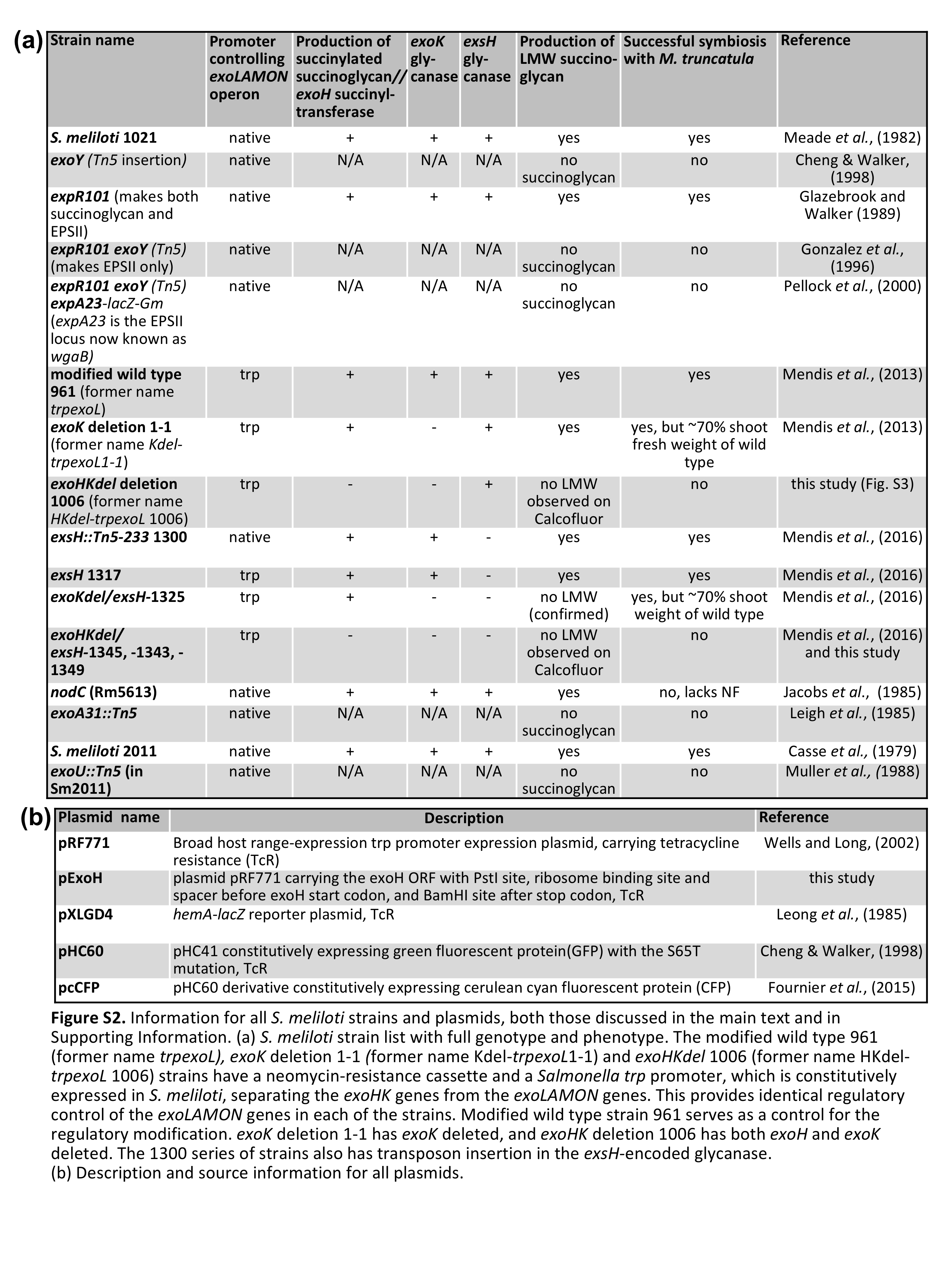

Supplement: Supplementary file 2 — Figure S2. Information for all S. meliloti strains and plasmids, both those are discussed in the main text and in Supporting Information. [file TPJ-102-311-s010.tiff]

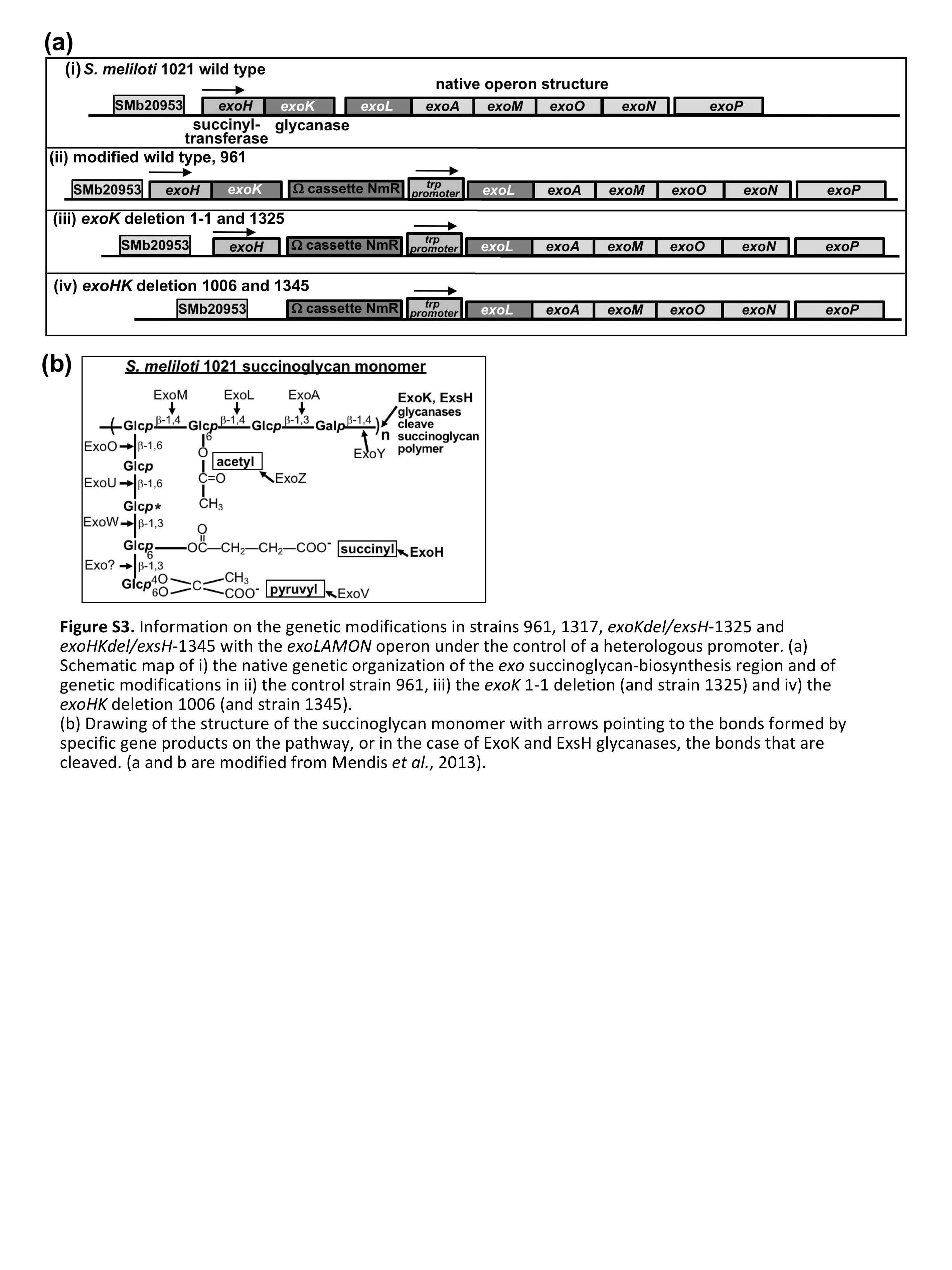

Supplement: Supplementary file 3 — Figure S3. Information on the genetic modifications in strains 961, 1317, exoKdel/exsH‐1325 and exoHKdel/exsH‐1345 with the exoLAMON operon under the control of a heterologous promoter. [file TPJ-102-311-s009.tiff]

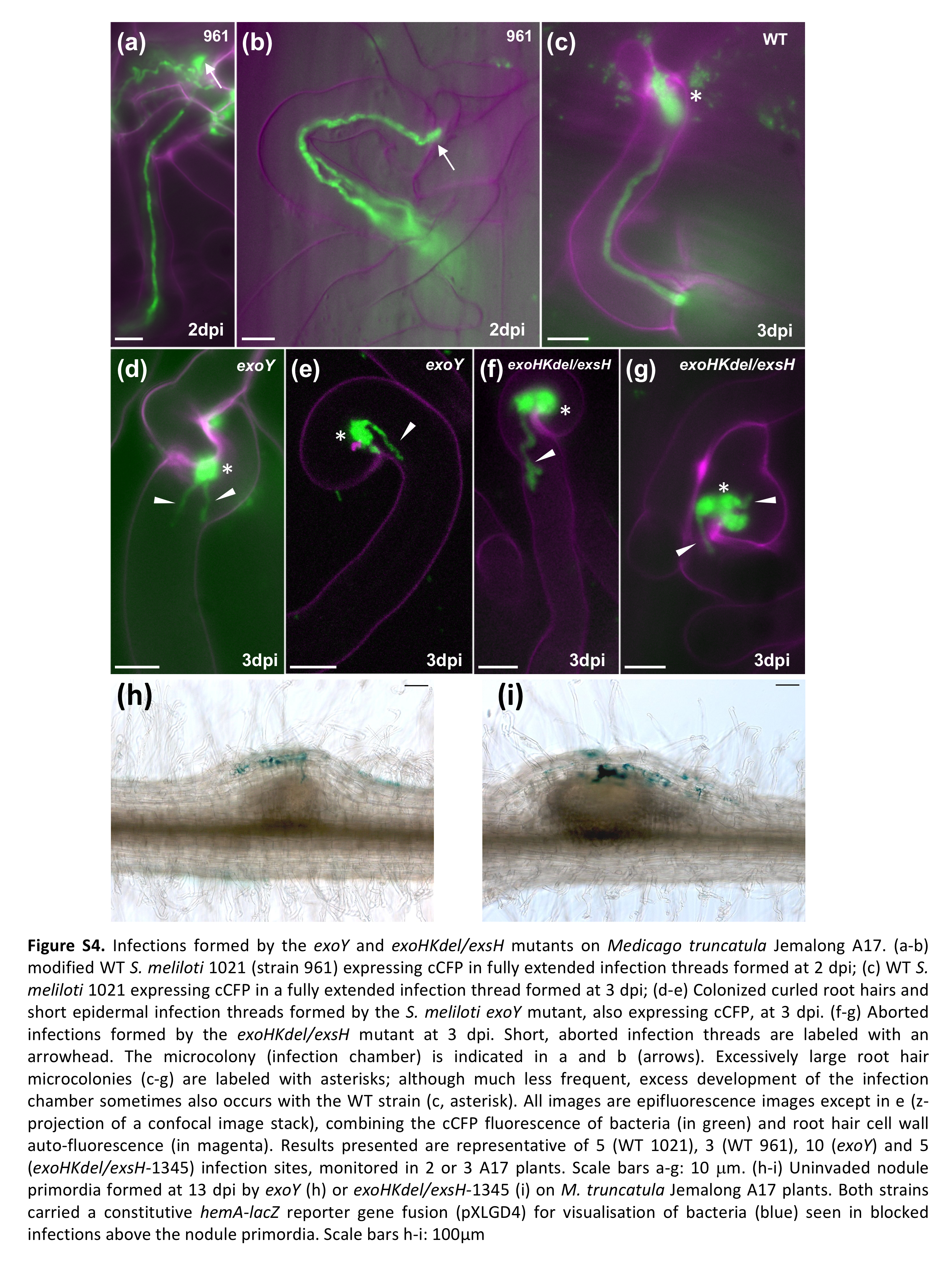

Supplement: Supplementary file 4 — Figure S4. Infections formed by the exoY and exoHKdel/exsH‐1345 mutants on Medicago truncatula Jemalong A17. [file TPJ-102-311-s008.tiff]

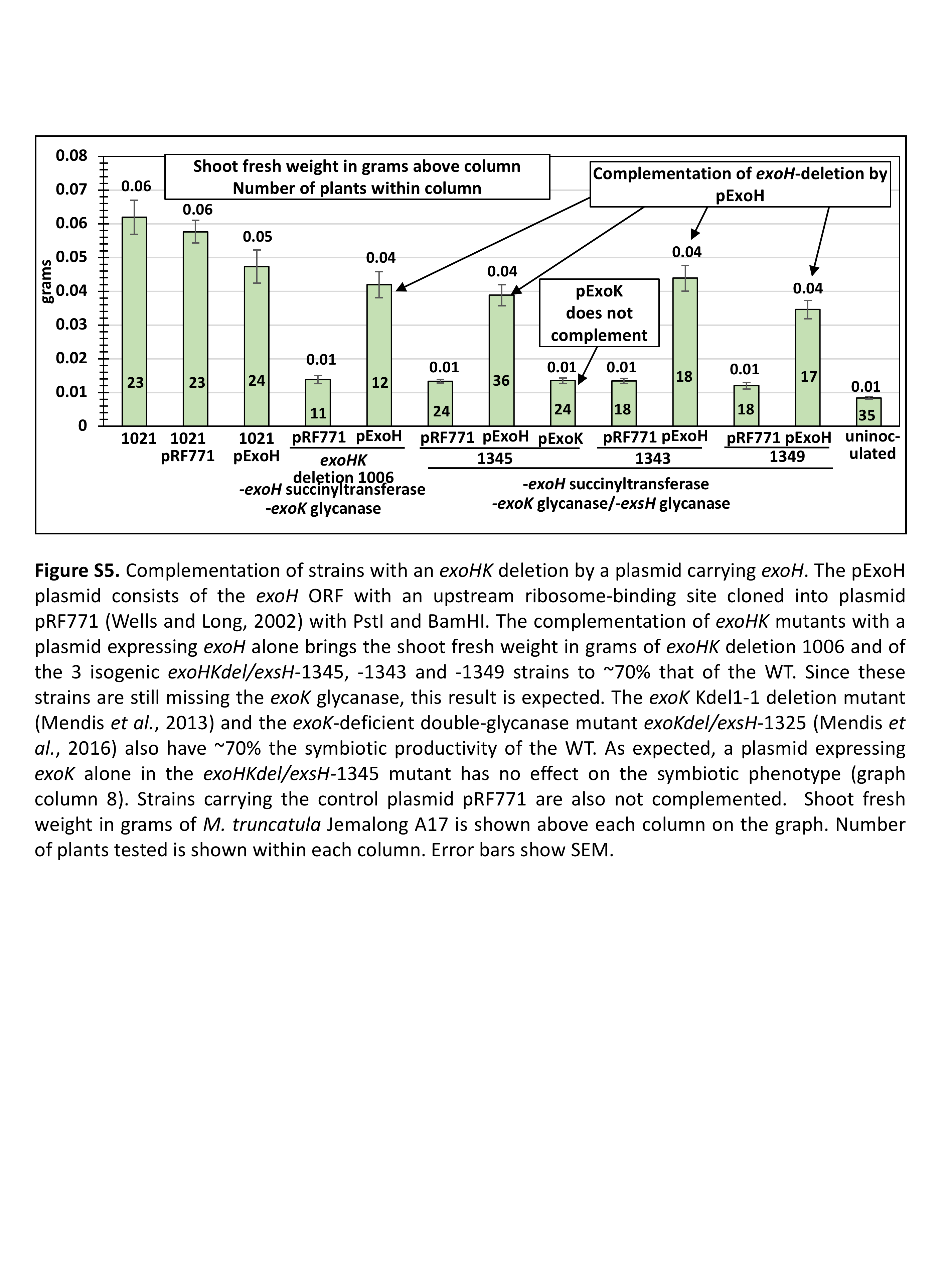

Supplement: Supplementary file 5 — Figure S5. Complementation of strains with an exoHK deletion by a plasmid carrying exoH. [file TPJ-102-311-s007.tiff]

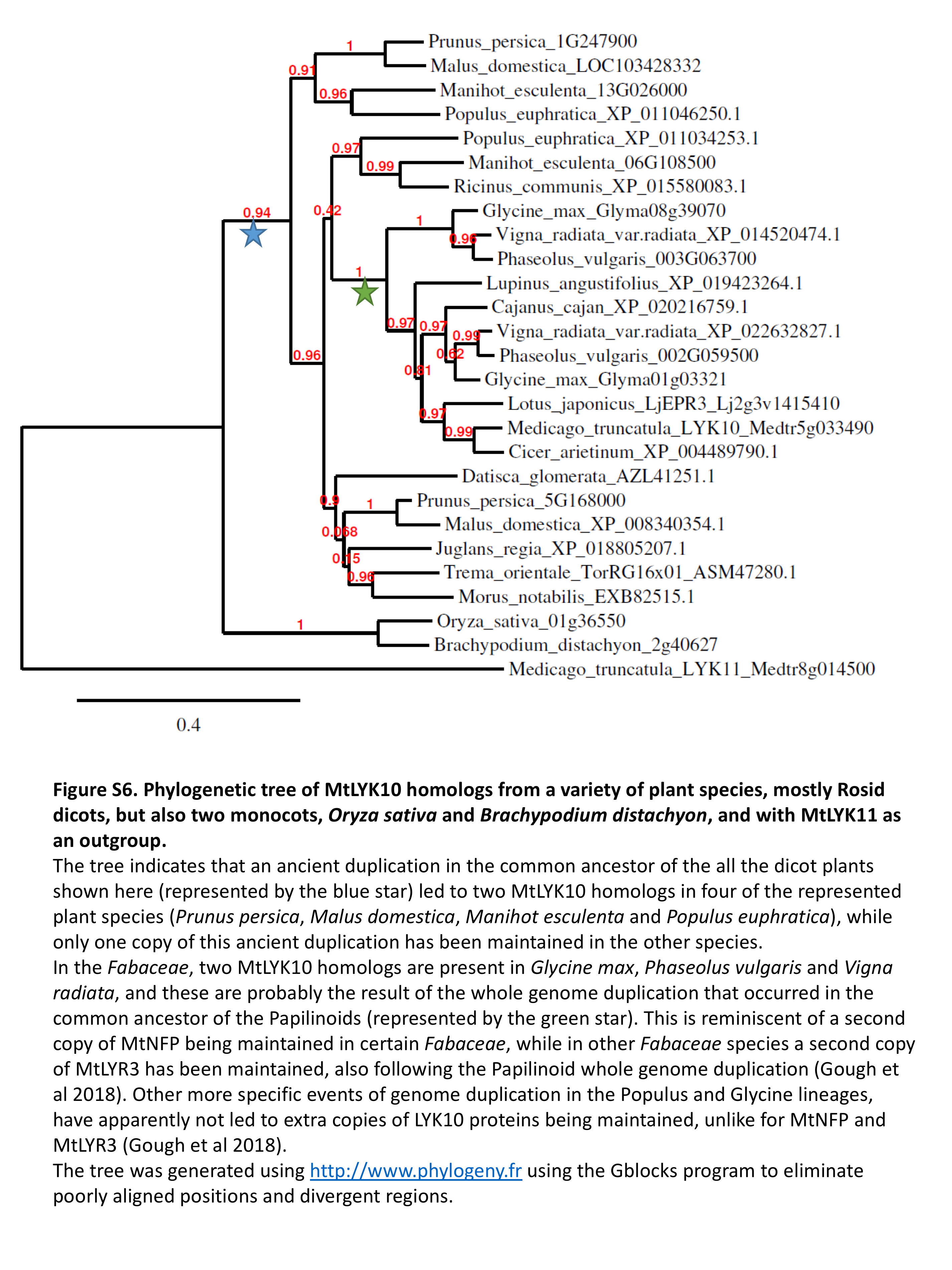

Supplement: Supplementary file 6 — Figure S6. Phylogenetic tree of MtLYK10 homologs from a variety of plant species, mostly Rosid dicots, but also two monocots, Oryza sativa and Brachypodium distachyon, and with MtLYK11 as an outgroup. [file TPJ-102-311-s011.tiff]

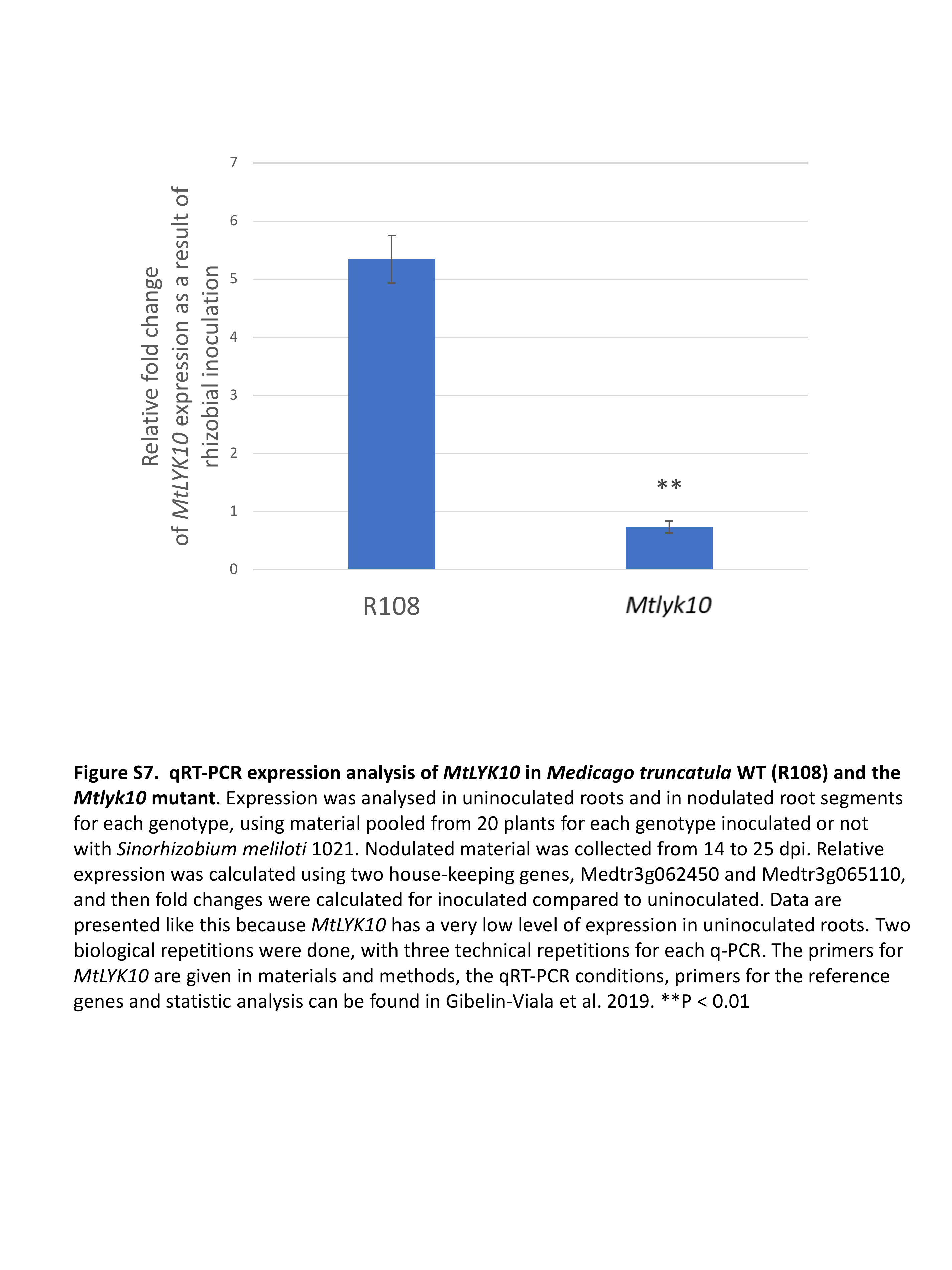

Supplement: Supplementary file 7 — Figure S7. qRT‐PCR expression analysis of MtLYK10 in Medicago truncatula WT (R108) and the Mtlyk10 mutant. [file TPJ-102-311-s001.tiff]

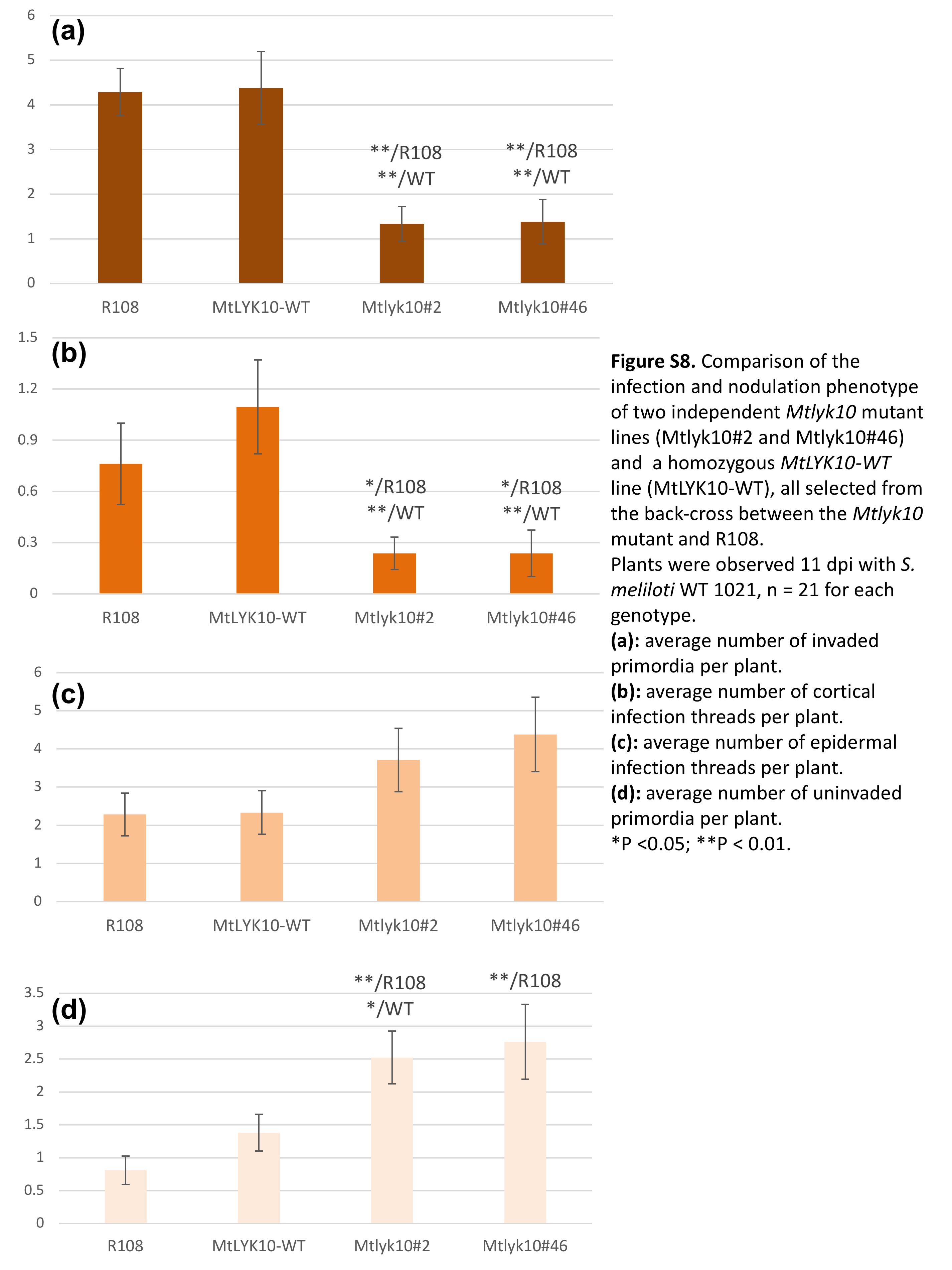

Supplement: Supplementary file 8 — Figure S8. Comparison of the infection and nodulation phenotype of two independent Mtlyk10 mutant lines (Mtlyk10#2 and Mtlyk10#46) and a homozygous MtLYK10‐WT line (MtLYK10‐WT), all selected from the backcross between the Mtlyk10 mutant and R108. [file TPJ-102-311-s002.tiff]

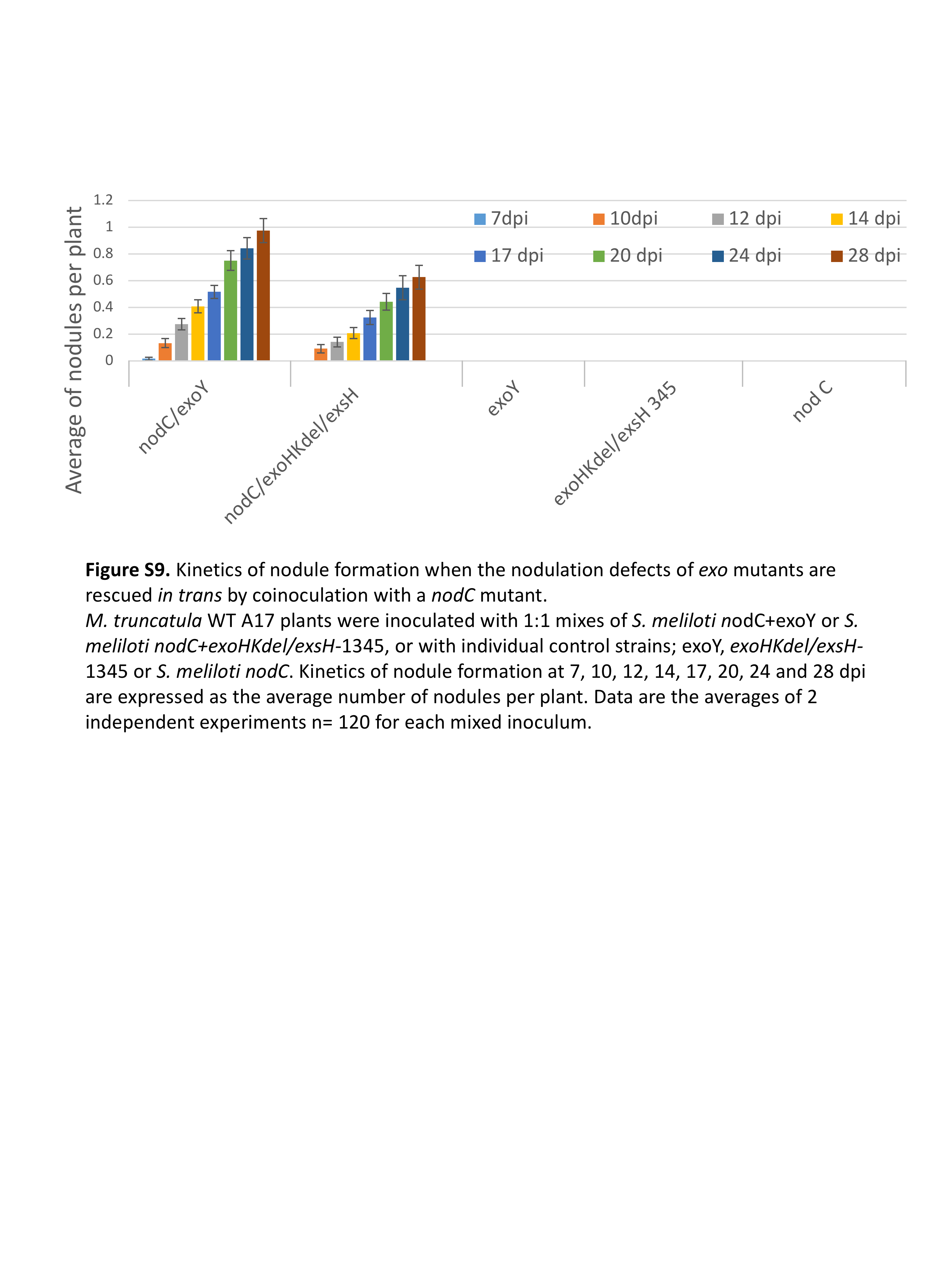

Supplement: Supplementary file 9 — Figure S9. Kinetics of nodule formation when the nodulation defects of exo mutants are rescued in trans by co‐inoculation with a nodC mutant. [file TPJ-102-311-s003.tiff]

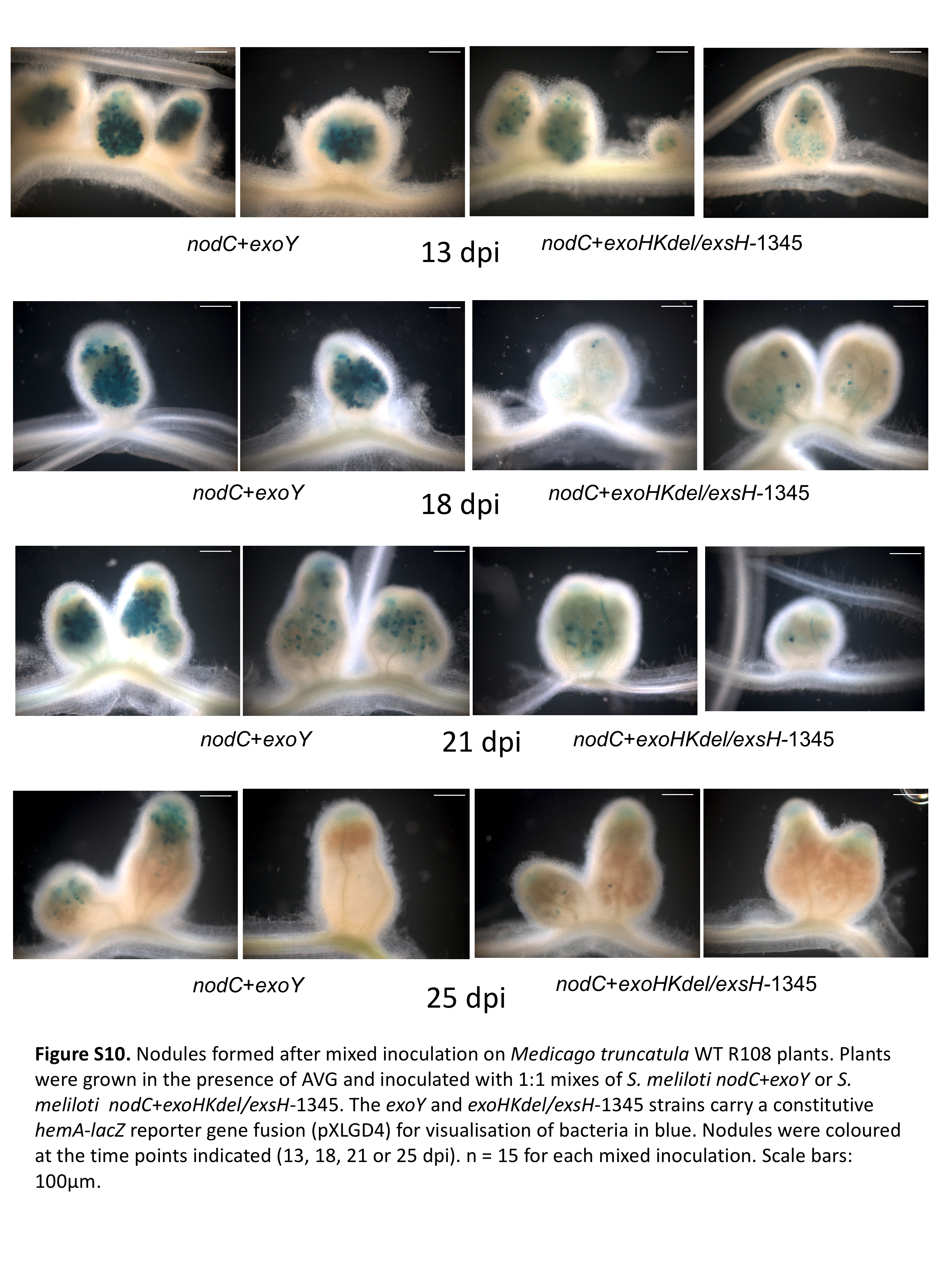

Supplement: Supplementary file 10 — Figure S10. Nodules formed after mixed inoculation on Medicago truncatula WT R108 plants. [file TPJ-102-311-s004.tiff]

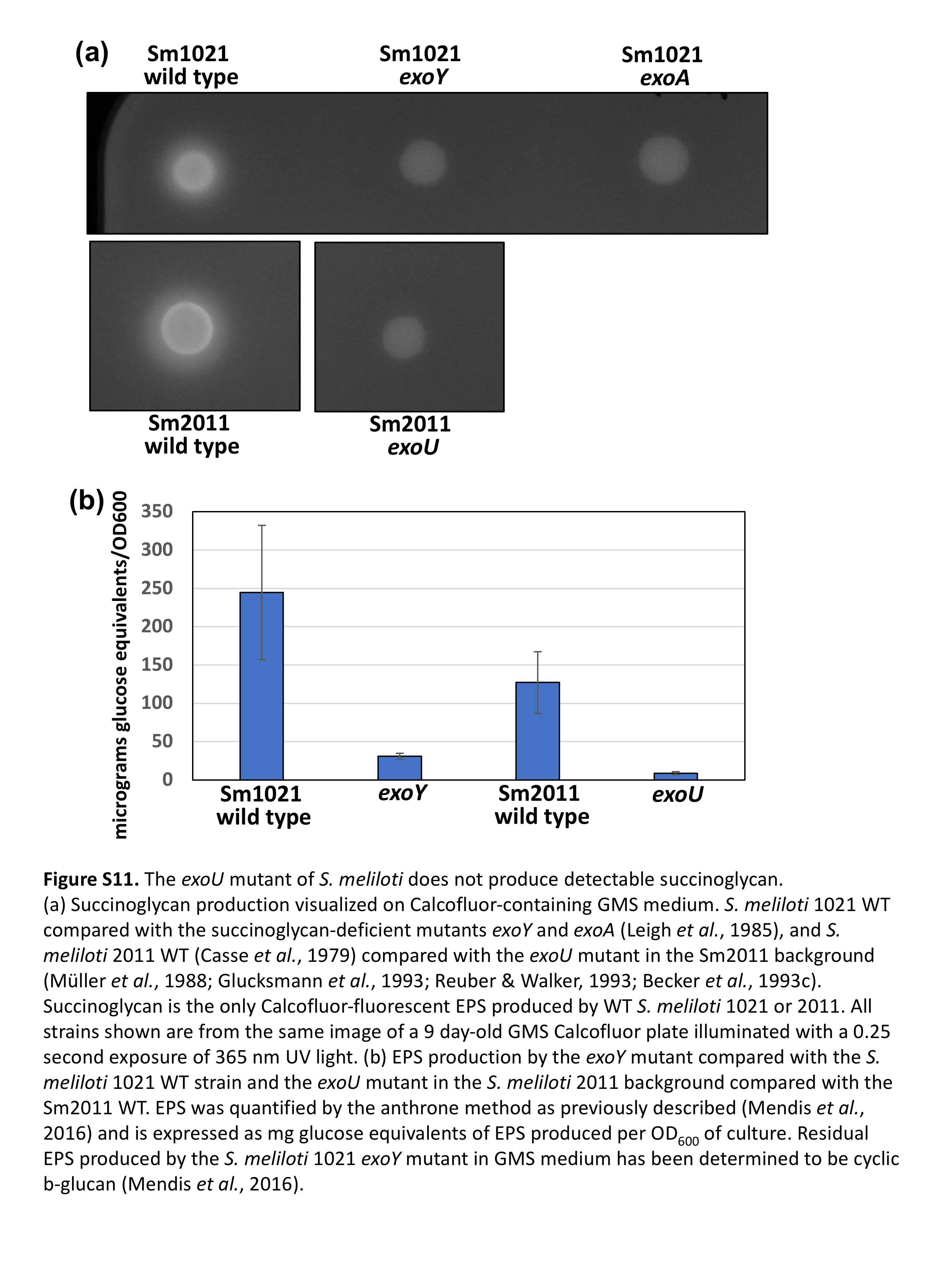

Supplement: Supplementary file 11 — Figure S11. The exoU mutant of S. meliloti does not produce detectable succinoglycan. [file TPJ-102-311-s005.tiff]
