# Supplementary material for: Sinorhizobium meliloti succinylated high‐molecular‐weight succinoglycan and the Medicago truncatula LysM receptor‐like kinase MtLYK10 participate independently in symbiotic infection
Source: Plant J. 2020 Jan 11;102(2):311–26. doi: 10.1111/tpj.14625 (PMC9327734; doi:10.1111/tpj.14625)
Supplement: Supplementary file 12 [file TPJ-102-311-s012.docx]

## *Plant Journal* Supporting Information

Article title: ***Sinorhizobium meliloti* succinylated high molecular weight succinoglycan and the *Medicago truncatula* LysM receptor-like kinase MtLYK10 participate independently in symbiotic infection**

Authors: Fabienne Maillet, Joëlle Fournier, Hajeewaka C. Mendis, Million Tadege, Jiangqi Wen, Pascal Ratet, Kirankumar S. Mysore, Clare Gough, Kathryn M. Jones

The following Supporting Information is available for this article:

**Figure S1.** Plant performance and nodulation phenotypes on alfalfa *cv.* Iroquois and *M. truncatula* A17 of *S. meliloti* strains that make EPSII (galactoglucan). (a) and (b) Plant performance measured as shoot length in cm and (c) and (d) number of fully-developed pink nodules shown for the same plants inoculated with the *S. meliloti* strain shown on the x-axis label. Data for alfalfa *cv.* Iroquois is shown in (a) and (c) and for *M. truncatula* A17 in (b) and (d). Unlike *S. meliloti* 1021 wild type, the *expR101* strain of *S. meliloti* is able to make EPSII. An *expR101 exoY* double mutant of *S. meliloti* makes EPSII, but does not make succinoglycan. On alfalfa Iroquois, this strain forms fully-developed nodules (c) and provides sufficient nitrogen for plant growth (a) on nitrogen-free BNM pH 6.5 medium. Nodulation mediated by EPSII is less efficient than that mediated by succinoglycan, resulting in less plant growth. A strain in the same genetic background as *expR101 exoY* that also has a *lacZ*-Gm insertion in the EPSII biosynthesis gene *expA23* (now known as *wgaB*) cannot make EPSII and does not form fully-developed, functional nodules on alfalfa. This demonstrates that EPSII can partially substitute for succinoglycan in nodule development on alfalfa. This was previously shown for alfalfa in Pellock *et al.,* 2000. In contrast, the EPSII-producing *expR101 exoY* strain does not form any fully-developed nodules on *M. truncatula* A17 (d), nor does it provide enough nitrogen for plant growth (b), compared to uninoculated plants. This demonstrates that EPSII cannot substitute for succinoglycan function in *S. meliloti* nodulation of *M. truncatula*. The number of plants tested is shown as a label on the graph bars in (a). Significant differences relative to the *S. meliloti* wild type 1021 and the *exoY* mutant are shown above the graph bars with ***P<0.001; **P<0.01; *P<0.05. A table describing these strains with source references is shown in Figure S2.

**Figure S2.** Information for all *S. meliloti* strains and plasmids, both those discussed in the main text and in Supporting Information. (a) *S. meliloti* strain list with full genotype and phenotype. The modified wild type 961 (former name *trpexoL*), *exoK* deletion 1-1 (former name *Kdel-trpexoL*1-1) and *exoHKdel* 1006 (former name *HKdel-trpexoL* 1006) strains have a neomycin-resistance cassette and a *Salmonella trp* promoter, which is constitutively expressed in *S. meliloti*, separating the *exoHK* genes from the *exoLAMON* genes. This provides identical regulatory control of the *exoLAMON* genes in each of the strains. Modified wild type strain 961 serves as a control for the regulatory modification. *exoK* deletion 1-1 has *exoK* deleted, and *exoHK* deletion 1006 has both *exoH* and *exoK* deleted. The 1300 series of strains also has transposon insertion in the *exsH*-encoded glycanase.

(b) Description and source information for all plasmids.

**Figure S3.** Information on the genetic modifications in strains 961, 1317, *exoKdel/exsH-*1325 and *exoHKdel/exsH-*1345 with the *exoLAMON* operon under the control of a heterologous promoter. (a) Schematic map of i) the native genetic organization of the exo succinoglycan-biosynthesis region and of genetic modifications in ii) the control strain 961, iii) the *exoK* 1-1 deletion (and strain 1325) and iv) the *exoHK* deletion 1006 (and strain 1345).

(b) Drawing of the structure of the succinoglycan monomer with arrows pointing to the bonds formed by specific gene products on the pathway, or in the case of ExoK and ExsH glycanases, the bonds that are cleaved. (a and b are modified from Mendis et al., 2013).

**Figure S4.** Infections formed by the *exoY* and *exoHKdel/exsH*-1345 mutants on *Medicago truncatula* Jemalong A17. (a-b) modified WT *S. meliloti* 1021 (strain 961) expressing cCFP in fully extended infection threads formed at 2 dpi; (c) WT *S. meliloti* 1021 expressing cCFP in a fully extended infection thread formed at 3 dpi; (d-e) Colonized curled root hairs and short epidermal infection threads formed by the *S. meliloti exoY* mutant, also expressing cCFP, at 3 dpi. (f-g) Aborted infections formed by the *exoHKdel/exsH* mutant at 3 dpi. Short, aborted infection threads are labeled with an arrowhead. The microcolony (infection chamber) is indicated in a and b (arrows). Excessively large root hair microcolonies (c-g) are labeled with asterisks; although much less frequent, excess development of the infection chamber sometimes also occurs with the WT strain (c, asterisk). All images are epifluorescence images except in e (z-projection of a confocal image stack), combining the cCFP fluorescence of bacteria (in green) and root hair cell wall auto-fluorescence (in magenta). Results presented are representative of 5 (WT 1021), 3 (WT 961), 10 (*exoY*) and 5 (*exoHKdel/exsH-*1345) infection sites, monitored in 2 or 3 A17 plants. Scale bars a-g: 10 μm. (h-i) Uninvaded nodule primordia formed at 13 dpi by *exoY* (h) or exoHKdel/exsH-1345 (i) on M. truncatula Jemalong A17 plants. Both strains carried a constitutive *hemA-lacZ* reporter gene fusion (pXLGD4) for visualisation of bacteria (blue) seen in blocked infections above the nodule primordia. Scale bars h-i: 100µm

**Figure S5.** Complementation of strains with an *exoHK* deletion by a plasmid carrying *exoH*. The pExoH plasmid consists of the *exoH* ORF with an upstream ribosome-binding site cloned into plasmid pRF771 (Wells and Long, 2002) with PstI and BamHI. The complementation of *exoHK* mutants with a plasmid expressing *exoH* alone brings the shoot fresh weight in grams of *exoHK* deletion 1006 and of the 3 isogenic *exoHKdel/exsH*-1345, -1343 and -1349 strains to ~70% that of the WT. Since these strains are still missing the *exoK* glycanase, this result is expected. The *exoK* Kdel1-1 deletion mutant (Mendis *et al.*, 2013) and the *exoK*-deficient double-glycanase mutant *exoKdel/exsH*-1325 (Mendis *et al.*, 2016) also have ~70% the symbiotic productivity of the WT. As expected, a plasmid expressing *exoK* alone in the *exoHKdel/exsH-*1345 mutant has no effect on the symbiotic phenotype (graph column 8). Strains carrying the control plasmid pRF771 are also not complemented. Shoot fresh weight in grams of *M. truncatula* Jemalong A17 is shown above each column on the graph. Number of plants tested is shown within each column. Error bars show SEM.

**Figure S6.** Phylogenetic tree of MtLYK10 homologs from a variety of plant species, mostly Rosid dicots, but also two monocots, *Oryza sativa* and *Brachypodium distachyon*, and with MtLYK11 as an outgroup. The tree indicates that an ancient duplication in the common ancestor of the all the dicot plants shown here (represented by the blue star) led to two MtLYK10 homologs in four of the represented plant species (*Prunus persica*, *Malus domestica*, *Manihot esculenta* and *Populus euphratica*), while only one copy of this ancient duplication has been maintained in the other species.

In the *Fabaceae*, two MtLYK10 homologs are present in *Glycine max*, *Phaseolus vulgaris* and *Vigna radiata*, and these are probably the result of the whole genome duplication that occurred in the common ancestor of the Papilinoids (represented by the green star). This is reminiscent of a second copy of MtNFP being maintained in certain *Fabaceae*, while in other *Fabaceae* species a second copy of MtLYR3 has been maintained, also following the Papilinoid whole genome duplication (Gough et al 2018). Other more specific events of genome duplication in the Populus and Glycine lineages, have apparently not led to extra copies of LYK10 proteins being maintained, unlike for MtNFP and MtLYR3 (Gough et al 2018).

The tree was generated using [http://www.phylogeny.fr](http://www.phylogeny.fr/) using the Gblocks program to eliminate poorly aligned positions and divergent regions.

**Figure S7.** qRT-PCR expression analysis of *MtLYK10* in *Medicago truncatula* WT (R108) and the *Mtlyk10* mutant. Expression was analysed in uninoculated roots and in nodulated root segments for each genotype, using material pooled from 20 plants for each genotype inoculated or not with *Sinorhizobium meliloti* 1021. Nodulated material was collected from 14 to 25 dpi. Relative expression was calculated using two house-keeping genes, Medtr3g062450 and Medtr3g065110, and then fold changes were calculated for inoculated compared to uninoculated. Data are presented like this because *MtLYK10* has a very low level of expression in uninoculated roots. Two biological repetitions were done, with three technical repetitions for each q-PCR. The primers for *MtLYK10* are given in materials and methods, the qRT-PCR conditions, primers for the reference genes and statistic analysis can be found in Gibelin-Viala et al. 2019. **P < 0.01

**Figure S8.** Comparison of the infection and nodulation phenotype of two independent *Mtlyk10* mutant lines (Mtlyk10#2 and Mtlyk10#46) and a homozygous *MtLYK10-WT* line (MtLYK10-WT), all selected from the back-cross between the *Mtlyk10* mutant and R108.

Plants were observed 11 dpi with *S. meliloti* WT 1021, n = 21 for each genotype.

(a): average number of invaded primordia per plant.

(b): average number of cortical infection threads per plant.

(c): average number of epidermal infection threads per plant.

(d): average number of uninvaded primordia per plant.

*P <0.05; **P < 0.01.

**Figure S9.** Kinetics of nodule formation when the nodulation defects of *exo* mutants are rescued *in trans* by coinoculation with a *nodC* mutant. *M. truncatula* WT A17 plants were inoculated with 1:1 mixes of *S. meliloti* *n*odC+exoY or *S. meliloti* *nodC+exoHKdel/exsH-*1345, or with individual control strains; *exoY*, *exoHKdel/exsH-*1345 or *S. meliloti* *nodC*. Kinetics of nodule formation at 7, 10, 12, 14, 17, 20, 24 and 28 dpi are expressed as the average number of nodules per plant. Data are the averages of 2 independent experiments n= 120 for each mixed inoculum.

**Figure S10.** Nodules formed after mixed inoculation on *Medicago truncatula* WT R108 plants. Plants were grown in the presence of AVG and inoculated with 1:1 mixes of *S. meliloti* *nodC*+*exoY* or *S. meliloti* *nodC*+*exoHKdel/exsH-*1345. The *exoY* and *exoHKdel/exsH-*1345 strains carry a constitutive *hemA-lacZ* reporter gene fusion (pXLGD4) for visualisation of bacteria in blue. Nodules were coloured at the time points indicated (13, 18, 21 or 25 dpi). n = 15 for each mixed inoculation. Scale bars: 100µm.

**Figure S11.** The *exoU* mutant of *S. meliloti* does not produce detectable succinoglycan.

(a) Succinoglycan production visualized on Calcofluor-containing GMS medium. *S. meliloti* 1021 WT compared with the succinoglycan-deficient mutants *exoY* and *exoA* (Leigh *et al.*, 1985), and *S. meliloti* 2011 WT (Casse *et al.*, 1979) compared with the *exoU* mutant in the Sm2011 background (Müller *et al.*, 1988; Glucksmann *et al.*, 1993; Reuber & Walker, 1993; Becker *et al.*, 1993c). Succinoglycan is the only Calcofluor-fluorescent EPS produced by WT *S. meliloti* 1021 or 2011. All strains shown are from the same image of a 9 day-old GMS Calcofluor plate illuminated with a 0.25 second exposure of 365 nm UV light. (b) EPS production by the *exoY* mutant compared with the *S. meliloti* 1021 WT strain and the *exoU* mutant in the *S. meliloti* 2011 background compared with the Sm2011 WT. EPS was quantified by the anthrone method as previously described (Mendis *et al.*, 2016) and is expressed as mg glucose equivalents of EPS produced per OD_600_ of culture. Residual EPS produced by the *S. meliloti* 1021 *exoY* mutant in GMS medium has been determined to be cyclic b-glucan (Mendis *et al.*, 2016).

**Supporting Information References**

**Becker A, Kleickmann A, Kuster H, Keller M, Arnold W, Puhler A. 1993c.** Analysis of the *Rhizobium meliloti* genes *exoU*, *exoV*, *exoW*, *exoT*, and *exoI* involved in exopolysaccharide biosynthesis and nodule invasion: *exoU* and *exoW* probably encode glucosyltransferases. *Molecular Plant-Microbe Interactions* **6:** 735-744.

**Casse F, Boucher C, Julliot S, Michel M, Dénarié J. 1979.** Identification and characterization of large plasmids in *Rhizobium meliloti* using agarose gel electrophoresis. *Journal of Bacteriology* **113:** 229-242.

**Cheng HP, Walker GC. 1998.** Succinoglycan is required for initiation and elongation of infection threads during nodulation of alfalfa by *Rhizobium meliloti*. *Journal of Bacteriology* **180:** 5183-5191.

**Fournier, J., Teillet, A., Chabaud, M., Ivanov, S., Genre, A., Limpens, E., de Carvalho-Niebel, F. and Barker, D.G. 2015.** Remodeling of the infection chamber before infection thread formation reveals a two-step mechanism for rhizobial entry into the host legume root hair. *Plant Physiology* **167:** 1233-1242.

**Gibelin-Viala C, Amblard E, Puech-Pages V, Bonhomme M, Garcia M, Bascaules-Bedin A, Fliegmann J, Wen J, Mysore KS, le Signor C, Jacquet C, Gough C. 2019.** The *Medicago truncatula* LysM receptor‐like kinase LYK9 plays a dual role in immunity and the arbuscular mycorrhizal symbiosis. *New Phytologist* **223:** 1516-1529.

**Glazebrook, J. and Walker, G.C. 1989.** A novel exopolysaccharide can function in place of the calcofluor-binding exopolysaccharide in nodulation of alfalfa by *Rhizobium meliloti*. *Cell* **56:** 661-672.

**Glucksmann MA, Reuber TL, Walker GC. 1993.** Family of glycosyl transferases needed for the synthesis of succinoglycan by *Rhizobium meliloti*. *Journal of Bacteriology* **175:** 7033-7044.

**Gonzalez, J.E., Reuhs, B.L. and Walker, G.C. 1996.** Low molecular weight EPS II of *Rhizobium meliloti* allows nodule invasion in *Medicago sativa*. *Proceedings of the National Academy of Sciences of the United States of America* **93:** 8636-8641.

**Gough C, Cottret L, Lefebvre B, Bono JJ. 2018.** Evolutionary history of plant LysM receptor proteins related to root endosymbiosis. *Frontiers in Plant Science* **9:** doi.org/10.3389/fpls.2018.00923

**Jacobs TW, Egelhoff TT, Long SR. 1985.** Physical and genetic map of a *Rhizobium meliloti* nodulation gene region and nucleotide sequence of *nodC*. *Journal of Bacteriology* **162:** 469-476.

**Leigh JA, Signer ER, Walker GC. 1985.** Exopolysaccharide-deficient mutants of *Rhizobium meliloti* that form ineffective nodules. *Proceedings of the National Academy of Sciences of the United States of America* **82:** 6231-6235.

**Leong, S.A., Williams, P.H. and Ditta, G.S. 1985.** Analysis of the 5' regulatory region of the gene for d-aminolevulinic acid synthetase of *Rhizobium meliloti*. *Nucleic Acids Ressearch* **13:** 5965-5976.

**Meade HM, Long SR, Ruvkun GB, Brown SE, Ausubel FM. 1982.** Physical and genetic characterization of symbiotic and auxotrophic mutants of *Rhizobium meliloti* induced by transposon *Tn5* mutagenesis. *Journal of Bacteriology* **149:** 114-122.

**Mendis HC, Madzima TF, Queiroux C, Jones KM. 2016.** Function of succinoglycan polysaccharide in *Sinorhizobium meliloti* host plant invasion depends on succinylation, not molecular weight. *mBio* **7**(3).

**Mendis HC, Queiroux C, Brewer TE, Davis OM, Washburn BK, Jones KM. 2013.** The succinoglycan endoglycanase encoded by *exoK* is required for efficient symbiosis of *Sinorhizobium meliloti* 1021 with the host plants *Medicago truncatula* and *Medicago sativa* (Alfalfa). *Molecular Plant-Microbe Interactions* **26:** 1089-1105.

**Müller P, Hynes M, Kapp D, Niehaus K, Pühler A. 1988.** Two classes of *Rhizobium meliloti* infection mutants differ in exopolysaccharide production and in coinoculation properties with nodulation mutants. *Molecular Genomics and Genetics* **211:** 17-26.

**Pellock, B.J., Cheng, H.P. and Walker, G.C. 2000.** Alfalfa root nodule invasion efficiency is dependent on *Sinorhizobium meliloti* polysaccharides. *Journal of Bacteriology* **182:** 4310-4318.

**Reuber TL, Walker GC. 1993.** Biosynthesis of succinoglycan, a symbiotically important exopolysaccharide of *Rhizobium meliloti.* *Cell* **74:** 269-280.

**Wells D. H. and Long, S. R. 2002.** The *Sinorhizobium meliloti* stringent response affects multiple aspects of symbiosis. *Journal of Bacteriology* **43:** 1115-27.
